# Explainable artificial intelligence for precision medicine in acute myeloid leukemia

**DOI:** 10.3389/fimmu.2022.977358

**Published:** 2022-09-29

**Authors:** Marian Gimeno, Edurne San José-Enériz, Sara Villar, Xabier Agirre, Felipe Prosper, Angel Rubio, Fernando Carazo

**Affiliations:** ^1^ Departamento de Ingeniería Biomédica y Ciencias, TECNUN, Universidad de Navarra, San Sebastián, Spain; ^2^ Programa Hemato-Oncología, Centro de Investigación Médica Aplicada, Instituto de Investigación Sanitaria de Navarra (IDISNA), Universidad de Navarra, Pamplona, Spain; ^3^ Centro de Investigación Biomédica en Red de Cáncer (CIBERONC), Madrid, Spain; ^4^ Departamento de Hematología and CCUN (Cancer Center University of Navarra), Clínica Universidad de Navarra, Universidad de Navarra, Pamplona, Spain; ^5^ Instituto de Ciencia de los Datos e Inteligencia Artificial (DATAI), Universidad de Navarra, Pamplona, Spain

**Keywords:** biomarkers, treatment selection, assignation problem, explainable artificial intelligence, drug repositioning, large-scale screening, ex-vivo experiment, drug sensitivity

## Abstract

Artificial intelligence (AI) can unveil novel personalized treatments based on drug screening and whole-exome sequencing experiments (WES). However, the concept of “black box” in AI limits the potential of this approach to be translated into the clinical practice. In contrast, explainable AI (XAI) focuses on making AI results understandable to humans. Here, we present a novel XAI method -called multi-dimensional module optimization (MOM)- that associates drug screening with genetic events, while guaranteeing that predictions are interpretable and robust. We applied MOM to an acute myeloid leukemia (AML) cohort of 319 *ex-vivo* tumor samples with 122 screened drugs and WES. MOM returned a therapeutic strategy based on the *FLT3*, *CBFβ-MYH11*, and *NRAS* status, which predicted AML patient response to Quizartinib, Trametinib, Selumetinib, and Crizotinib. We successfully validated the results in three different large-scale screening experiments. We believe that XAI will help healthcare providers and drug regulators better understand AI medical decisions.

## 1 Introduction

The advance of personalized medicine, and in particular precision oncology, is partially based on the development of drug sensitivity studies. These experiments are promoting the discovery of new drugs, biomarkers of sensitivity, and drug repositioning. With increasing frequency, these studies have widened their scope from single drug studies to experiments involving hundreds of drugs, also known as drug screening. In recent years, drug screenings are being carried out on hundreds of cell lines giving rise to large-scale drug screening datasets, e.g., GDSC, which includes 130 screened drugs in an average of 368 lines per drug (1). Combining these drug sensitivity studies with tumor genotypes makes it possible to associate the response to treatment with genetic alterations (biomarkers), thus promoting the search for new personalized therapies (2).

Exploring the potential of these experiments, artificial intelligence (AI) algorithms for personalized medicine focus on the analysis of such datasets to bridge the gap for drug discovery. Some studies use machine learning algorithms for monotherapy prediction (3, 4), other approaches are based on training deep learning (DL) models from patients’ omics data (5, 6). These methods create black-box predictors that make agnostic inferences of treatment for a patient based on complex non-linear relationships. The output is, for these cases, an individual therapy for a patient, instead of a general treatment guideline (7). Despite optimizing patient treatment, this approach has the inherent disadvantages of methods based on neural networks: they require a huge amount of data, and therefore experiments are unable to show the criteria that trigger the decision –since neural networks tend to be black-box models–. These technical challenges are limiting the translation of drug screening experiments to clinical practice.

Explainable Artificial Intelligence (XAI) focuses on making AI understandable to humans by the usage of “white-box” algorithms that allow end-users to understand why the model predicts a certain solution (8, 9). The importance of using XAI models in the finding of new personalized treatments is twofold: therapeutic pipelines can be more easily adopted in normal clinical guides (e.g., using a decision tree that does not require a complex model with a high number of variables) (9) and drug regulators, such as the Food and Drug Administration (FDA), or European Medicines Agency (EMA) will have an easier journey to approve a drug if the companion biomarkers are reasonable and robust (10, 11). Consequently, XAI opens the door to bridge the gap between clinical practice and bioinformatics (8, 12).

In this study we have developed a new XAI method, called multi-dimensional module optimization (MOM) algorithm, to predict therapeutic strategies based on large-scale drug screening data. This method systematically associates drugs with combined sets of genetic biomarkers that can be generalized and applied to other cohorts of patients. The therapeutic strategies provided by MOM can easily be understood by humans and are easy to implement in the clinical practice with a process equivalent to a decision tree. The optimization problem considers the effect of drug toxicity focusing on providing drugs that are differentially effective to patients with a specific genotype. MOM’s result is deterministic −this is important to get regulatory approvals− and guaranteed to be optimal, each patient is given the best possible treatment.

We selected Acute Myeloid Leukemia (AML) as a disease model, a highly heterogeneous type of cancer that affects bone marrow cell precursors. In AML, genomic profiling is essential to understand its biology, diagnosis, and treatment (13–15). Unfortunately, 70% of adult people diagnosed with this disease die within five years of diagnosis (16). The current ELN (European Leukemia Network) risk stratification is based on the genetic biomarkers of the disease (17). Although there are big prognosis differences across these genetic groups, the current approach for young and fit patients is a standard induction cytotoxic therapy (“3+7”) (14, 17) with the addition of targeted therapies, mainly *FLT3* inhibitors, to a specific group of AML patients (14). Despite 8 new drugs have been approved for AML in the last years, its lethality is still very high. In addition, there are no targeted treatments directed to *FLT3^WT^* patients –70% of all AML cases (18). A machine learning approach that identifies the most adequate FLT3 inhibitor as well as the treatment for other AML genotypes, would allow the discovery of new indications for other drugs for the AML. As a result, a new classification guide based on the response to therapy for specific genetic alterations would be beneficial in clinical practice.

We applied MOM to the BeatAML project cohort, which carried out WES (Whole Exome Sequencing) and drug screening experiments of 122 drugs with *ex-vivo* AML tumor samples from 319 patients (19). Ex-vivo experiments in hematological cancers are of great importance since they are performed directly on the patient’s living tumor cells (19, 20), allowing to correlate drug sensitivity to the patient’s genotype. The results obtained using MOM are *in-silico* validated using K-fold cross-validation and in three independent large-scale experiments, one based on pan-cancer drug sensitivity and two referred to pan-cancer gene essentiality using siRNA and *CRISPR*-cas9. MOM’s patient indications require only three different biomarkers, which makes them to be easily understood by the clinician.

## 2 Results

### 2.1 An explainable artificial intelligence method to predict optimal treatments based on patient genotype

The implementation of a clinical translational XAI model requires the development of a robust method to associate biomarkers to specific targeted treatments. and, thus, relating drug sensitivity and patient genetic events -including SNVs, indels, fusion genes, or even epigenetics. The development of an AI algorithm in this context requires to solve three important challenges: (i) proper modeling of the toxicity of screened drugs (most aggressive drugs are not necessarily better treatments), (ii) dealing with a high number of statistical hypotheses that intrinsically increase false discovery rate (FDR), and (iii) explaining the internal reasoning that the model uses to propose a decision so that it is easy to approve and implement in the clinical practice.

**Figure 1:**
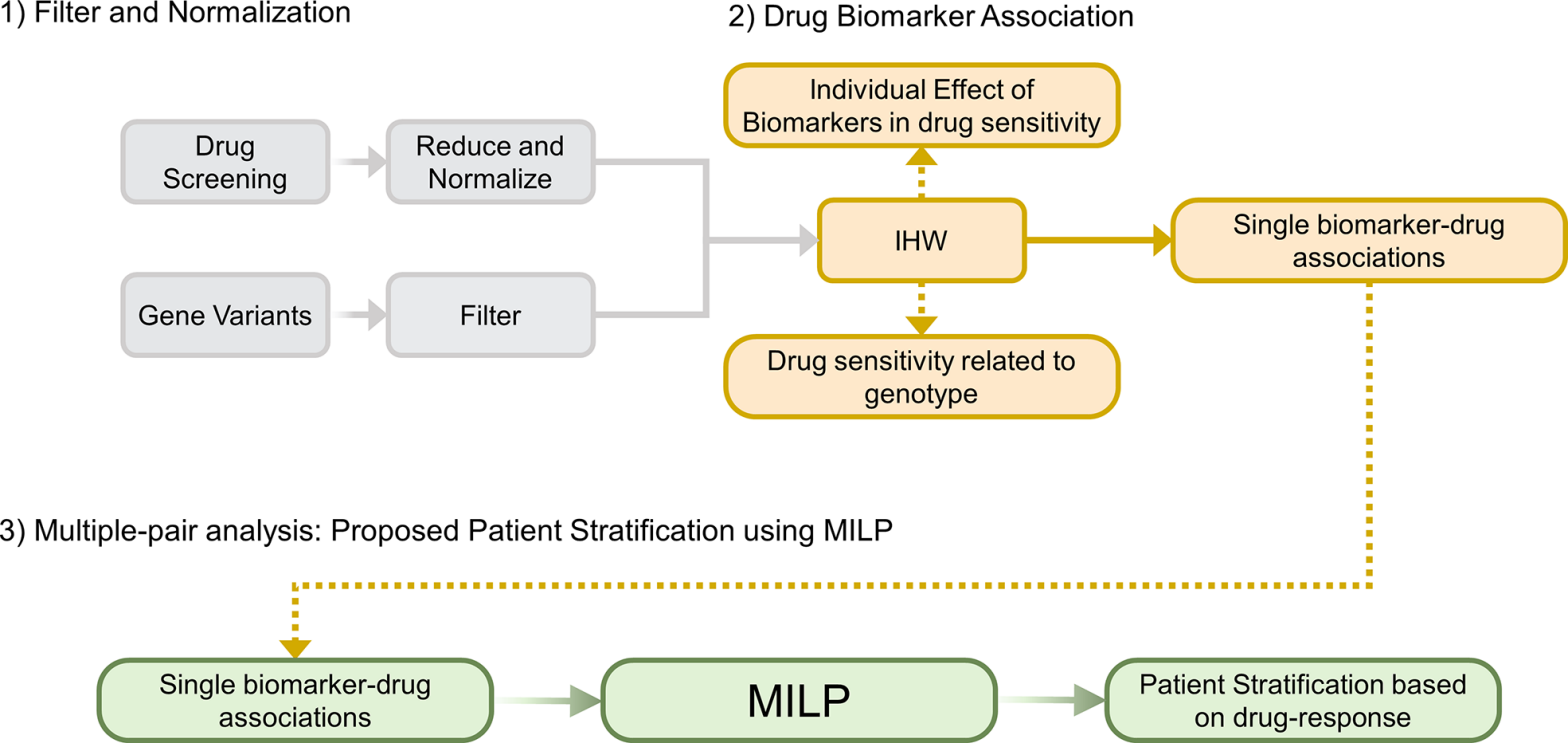
Overview of MOM’s pipeline. **(1)** Filter and Normalization. **(2)** Generate individual Drug-Biomarker Associations using IHW, **(3)** Multiple-pair analysis that generates a patient stratification guideline using a novel MILP model (IHW: Independent Hypothesis Weighting, MILP: Mixed-integer Linear Programming).

We propose an algorithm named Multi-dimensional Module Optimization (MOM) that addresses each of these challenges by dividing the problem into three main steps (**Figure 1**): preprocessing the input drug sensitivity scores, associating single biomarkers to drugs with an increased statistical power and combining individual treatments to unveil multi-step treatment pipelines to stratify patients based on drug-response.

MOM is developed to optimally stratify patients following a decision tree based on simple logical rules, in which each step is defined by the presence or absence of a certain biomarker and the recommendation of one drug. In turn, MOM requires genetic variants information and drug sensitivity screenings as input data.

**Figure 2:**
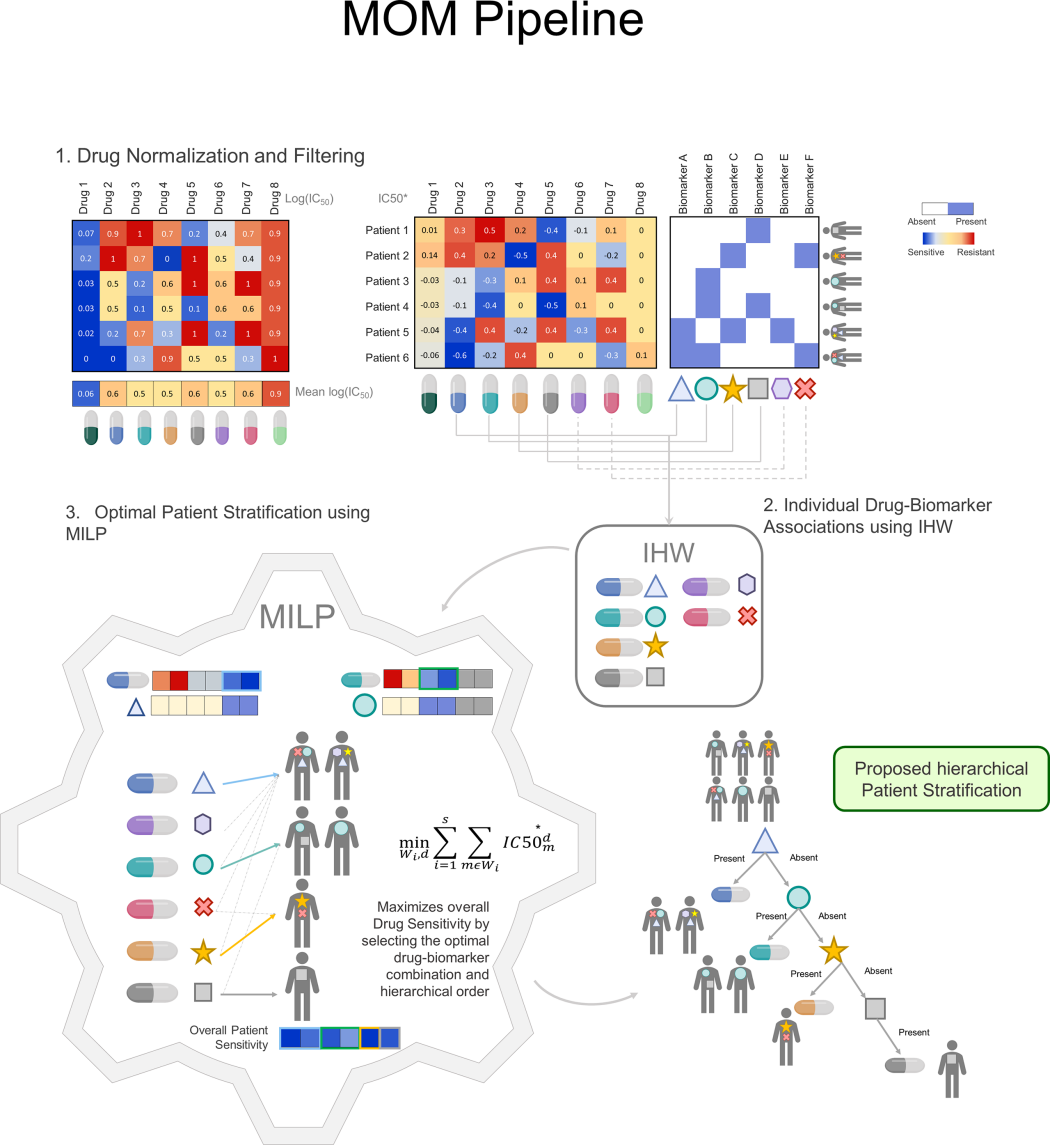
MOM Pipeline: MOM pipeline is defined by 3 major steps: **1**) Drug normalization to reduce drug toxicity. It is performed by removing drug mean effect in all patients. The blue color represents drug sensitivity for the sample whereas the red color represents drug resistance in the sample. **2)** Individual Drug biomarker associations using IHW. Drugs are matched to biomarkers profile, all individual associations generate a p-value that is corrected using IHW. IHW selects the candidate biomarkers and treatment and are used as input to the MILP problem. **3**) Optimal Patient Stratification using MILP. The MILP module receives as input the normalized drug information IC50* and the candidate individual associations and outputs a decision tree for clinical decision-making guidance. Within this module, the treatment is optimized so that each patient receives the drug for which is more sensitive. (IHW: Independent Hypothesis Weighting, MILP: Mixed-Integer linear Programing).

To illustrate the steps of the algorithm, let us consider a toy example with 8 drugs and their corresponding drug-response scores for 6 patients (**Figure 2**). In this case, as in every precision medicine scenario, we want to find robust companion biomarkers that, associated to drugs allow us to maximize patient response with minimized toxicity.

In the first step, MOM preprocesses drug sensitivity scores (**Figure 2.1**). For which, instead of using the standard measure of IC_50_, we proposed an incremental version of the logarithm of the IC_50_, named IC50* (See Methods for more details). The proposed correction has two main advantages. First, MOM prioritizes drugs that have a differential effect on different patients, which are, in turn, better candidates to develop a personalized treatment based on a companion biomarker. Second, drugs whose effectiveness does not depend on patient genotype are more unspecific and, therefore, more prone to be toxic for different tissues. In the next section, we will illustrate this fact with a real case scenario.

To exemplify this normalization, let us return to the toy example with 6 patients, 8 drugs and their corresponding log(IC_50_) scores measured in ex-vivo tumors (**Figure 2.1**). Considering raw log(IC_50_) exclusively (left-hand heatmap), it could be argued that Drug 1 is the most effective drug and, therefore, it should be indicated to all patients regardless their genotype. However, since the dose can be adjusted for each patient, Drugs 1 and 8 will be given at a small and a large dose respectively balancing their effect. Using IC50* (right-hand panel) allows MOM to maximize the genetic dependence of drugs, rather than the absolute cellular death in patient tumors.

In the second step (**Figure 2.2**), MOM provides single biomarker-treatment associations by prioritizing the drugs whose response is associated with patient genotype. The selected statistical analysis to find the biomarker-treatment associations is the Independent Hypothesis Weighting (IHW) algorithm. This algorithm has been proved to increase the power of tests in several biological scenarios (21, 22).

This algorithm provides also two interesting “by-products”: i) identifies which biomarkers are related to drug sensitivity, e. gr. *TP53* is usually a source of resistance, ii) identifies drugs whose efficacy is related to the genetic profile, Olaparib is effective only for *BRCA* ^Mut^ patients (23).

In the third step (**Figure 2.3**), MOM predicts a sequential treatment guideline that maximizes the drug effect on the group of patients that share the genotype of the selected biomarkers. Using Mixed integer Linear Programing (MILP)(see **Supplementary Methods**), MOM gets the optimal treatment guideline (decision tree). MILP is a versatile optimization method that allows the solution of complex mathematical problems using integer variables and assures that the drug assignation is optimal. This solution (i) is explainable (XAI); (ii) eases the translation into clinical practice; and (iii) assures a global and deterministic optimum to the problem.

### 2.2 *FLT3*, *CBF*β*-MYH11*, and *NRAS* variants play a key role in acute myeloid leukemia sensitivity to quizartinib, trametinib, and selumetinib

We selected the BeatAML cohort to test MOM as it contains *ex-vivo* drug sensitivity screenings of 122 drugs in AML tumors derived from 319 patients (19), and includes both whole-exome sequencing experiments (WES) and drug sensitivity for every patient. This cohort, allows us to measure the impact of genetic variants on drug sensitivity (**Supplementary Figures 13-19**). In addition, AML is a good disease model to develop precision treatments, as it is a highly heterogeneous disease in which genomic profiling is essential to understand its biology, diagnosis, and treatment (13–15). Patients within this cohort are in different therapeutic stages, e.g., induction, maintenance, consolidation, or palliative care (among others), there also are 32 *de novo* patients (**Supplementary Figure 12**).

The drugs studied in the BeatAML cohort cover a wide variety of different cancers and diseases: 24% are indicated for AML, 16% for other leukemias types, 10% for multiple myeloma, and 4% for lymphomas. This means that 54% of the drugs have been studied for hematological malignancies. The rest 46% include drugs used in lung, breast, or renal cancers among other diseases (**Supplementary Figure 20**). Focusing on AML, the dataset provides a total of 11 AML drugs already in clinical use -e.g. Venetoclax, Quizartinib, or Gilteritinib- and 18 AML experimental drugs -e.g. Panobinostat, Lestaurtinib, or Pazopanib.

We filtered gene variants to keep the ones that appear in at least 4 out of 319 patients (1%). This process provides 64 potential single biomarkers. We also removed drugs used in less than 20% of the patients, and those without a candidate gene target. After matching samples with *ex-vivo* and WES experiments, we finally get the *ex-vivo* screening of 111 drugs for 319 patients (see Methods for more details). We then applied the MOM algorithm to this cohort to unveil groups of AML patients that share genotype and drug sensitivity. In the first step, MOM normalizes the IC_50_ values to define a score that better defines tumor sensitivity, namely IC50*.

**Figure 3:**
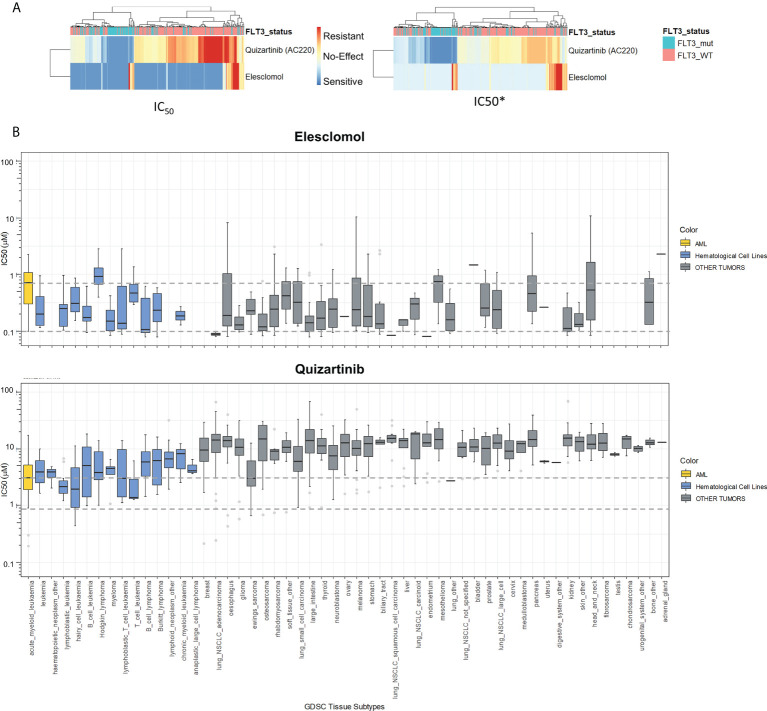
IC_50_ Normalization to Avoid Drug Toxicity. **(A)** Drug Sensitivity Heatmap in BeatAML cohort. The left panel shows the IC_50_ values for AML tumors of BeatAML. Effectiveness of a drug in a patient is plotted in blue color, and resistance is represented in red color. The right panel shows the sensitivity in IC50* score. **(B)** Drug sensitivity of Quizartinib and Elesclomol across different tissue types using GDSC. IC_50_ values relative to different tissues are shown in the graph. In yellow color are plotted the sensitivity values of AML cell lines, in blue color are plotted the drug sensitivity values for the Hematological cell lines, and finally in grey color, are plotted the sensitivity values for the non-hematological tissues from GDSC. Dotted grey lines represent the second IC_50_ quantile for AML cell lines (GDSC: Genomics of Drug Sensitivity in Cancer). IC50* is the name of our new sensitivity score.

Let us illustrate this with a paradigmatic example. In our dataset, the median IC_50_ for Elesclomol is much smaller than the median IC_50_ for Quizartinib (**Figure 3A**, left panel). Consequently, Elesclomol seems a better option to treat patients with AML. **Figure 3B** gives a completely different reading: Elesclomol is more toxic in almost any tissue if compared with the AML lines. On the contrary, Quizartinib is more toxic on AML than in most other tissues. This simple example shows that plain IC_50_ must not be used to select the treatment guideline for the patients. The higher value of IC_50_ for Quizartinib could be corrected by adjusting the dose. In **Figure 3A**, right-panel, after the normalization, the IC_50_* for Elesclomol appears less effective, whereas Quizartinib preserves its sensitivity profile, which, in this example, it is related to the *FLT3* status of the tumor.

**Figure 4:**
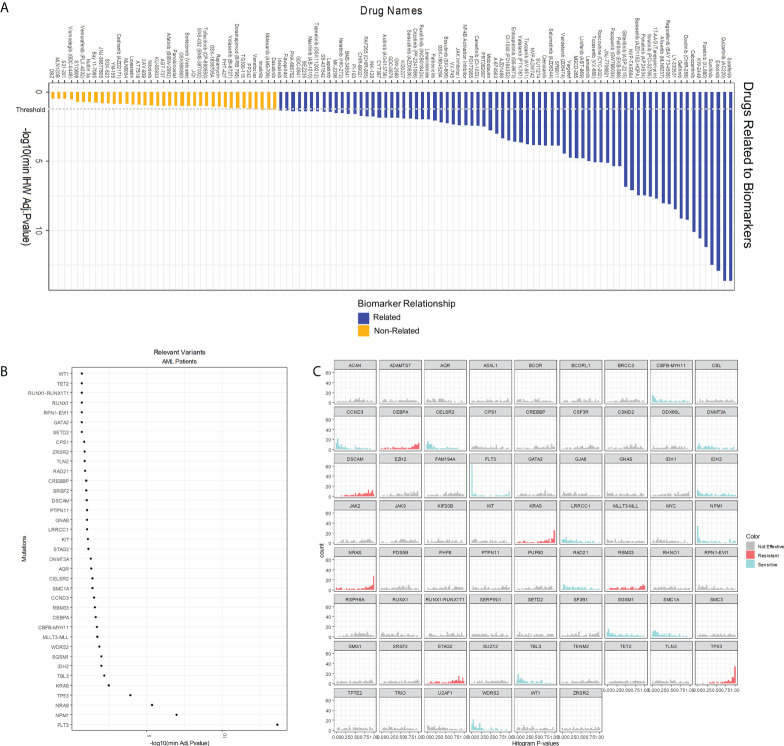
Analysis of single interactions biomarker-drug. **(A)** Overall score of 122 drugs whose IC50* is related or non-related to cell genotype according to our model. A drug is related to a relevant variant (those whose IHW weight is greater than zero) if its adjusted p-value is below 0.05. **(B)** Global effect of AML gene variants in AML drug sensitivity. The x-axis shows the logarithm of the minimum adjusted p-value of the biomarker with any of the drugs. Only those biomarkers whose IHW weight is greater than zero are shown. **(C)** One-tail p-value histograms comparing drug sensitivity according to the biomarker status in AML. If a histogram has a strong peak near zero, patients with the biomarker are sensitive to many drugs. On the contrary, if a histogram has a strong peak near one, patients with the biomarker are resistant to many drugs. A genetic variant is considered to confer sensitiveness if the number of drugs whose p-value<0.2 is twice the number of p-values >0.2. Similarly, a variant confers resistance if fulfills that the number of p-values>0.8 is twice the number of p-values<0.8. (IHW: Independent Hypothesis Weighting).

In the second step, MOM calculates individual associations between drugs and genetic alterations using the IHW strategy (21). This approach sheds light on which drugs can be influenced by patient genotype (**Figure 4A**). IHW also provides a weight for each genetic variant related to the probability of such variant to be a true positive. Non-zero IHW weights represent genetic variants that reduce the FDR and increase the power of tests as demonstrated by IHW authors (21). IHW estimates that, in our AML cohort, 37 biomarkers have weights greater than zero. IHW weights can be therefore used to state the relevance of each biomarker. We sorted IHW weights confirming that *FLT3^Mut^*, *NPM1^Mut^*, *NRAS^Mut^*, *TP53^Mut^*, and *KRAS^Mut^* are included in the top 5 biomarkers (**Figure 4B**), which have already been described in previous studies (24–29). IHW also provides an adjusted p-value for each drug-biomarker association. For instance, the pipeline identified the known relation of *FLT3* internal tandem duplications (*FLT3-ITD*) patients being more sensitive to Sorafenib, Quizartinib, or Gilteritinib (**Supplementary Table 1**; **Supplementary Figure 21**).

Interestingly, an indirect output of this second MOM step is the quantification of the sensitiveness or resistance triggered by a specific genetic variant. Summarizing this score, gene variants can be classified by their effect: either sensitive or resistant to the tested drugs (**Figure 4C**). For example, variants in *FLT3* or *NPM1* are associated with a more sensitive response for the cohort of drugs in this experiment, whereas genetic alterations in *KRAS*, *NRAS*, or *TP53* are more likely resistance-conferring. Other results include *CCND3*, *WDR52*, *CELSR2*, *CBFβ*-*MYH11*, and *SMC1A* as biomarkers of sensitivity and *STAG2* of resistance. This effect is relative to the studied dataset, Beat AML, and occurs across 66 different drugs studied or prescribed for hematological malignancies.

**Figure 5:**
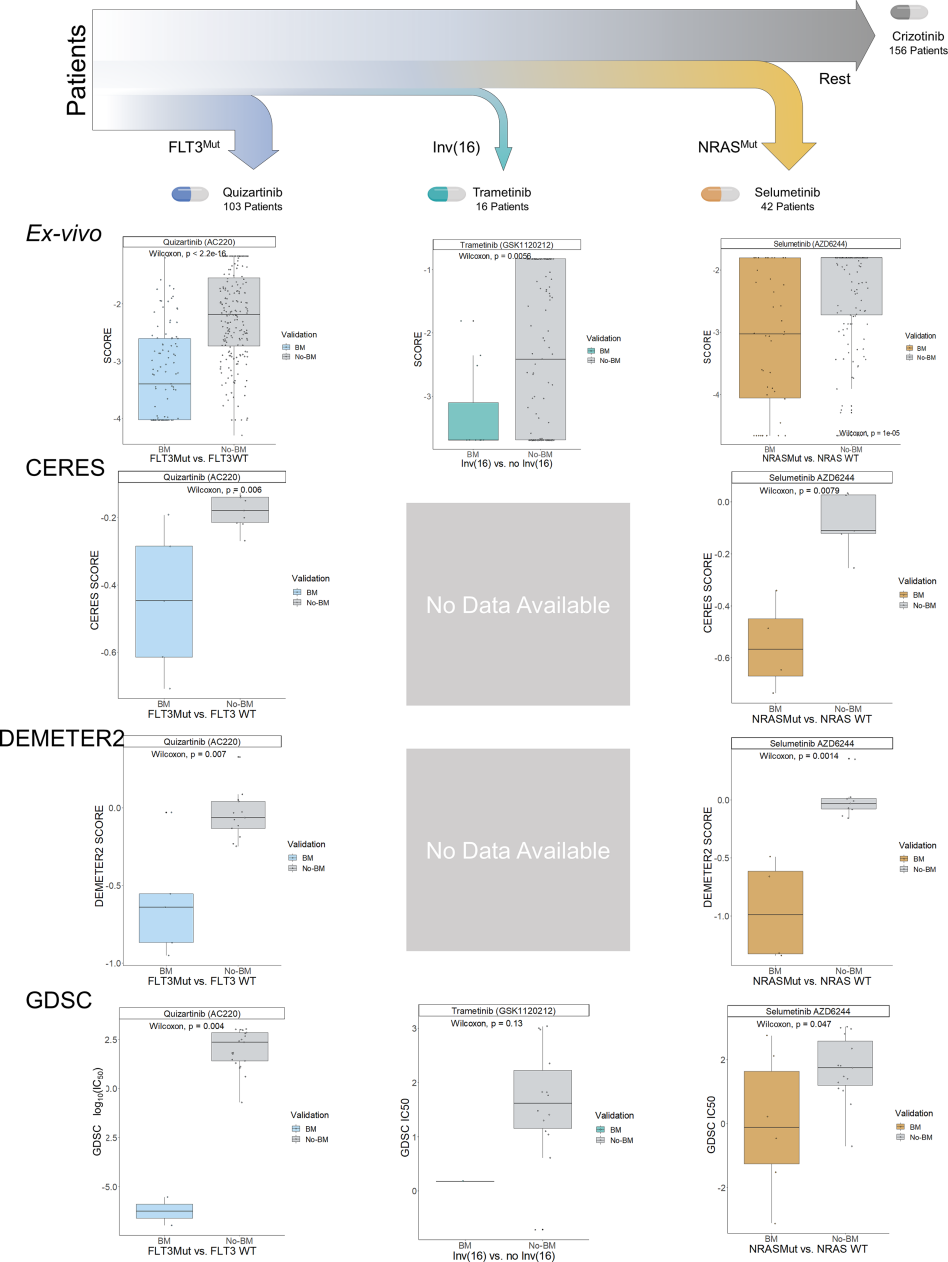
Decision Tree for the Proposed Patient Stratification using MOM. MILP from MOM obtained a hierarchical clinical guideline for patient stratification consisting of 4 different subgroups. Each of them is denoted by a biomarker and represented by color (blue, turquoise, orange, and grey). These subgroups were validated in the BeatAML ex-vivo cohort, CERES, DEMETER2, and GDSC. Boxplots show the results of the validation. The y-axis represents the essentiality score from the different experiments and the x-axis represents the biomarker presence-absence of the samples. The validation was performed sequentially, already treated samples from previous subgroups were excluded in the following subgroups i.e. samples with *FLT3^Mut^* (blue) from the first boxplot are not plotted in the non-biomarker (grey) in the second boxplot. CERES and DEMETER2 do not have experiments with cell lines having inv(16).

Finally, in the third step, we solved the MILP problem from MOM using the individual candidate associations. As a result, MOM returns a decision tree that, depending on the presence or absence of several biomarkers, recommends a treatment for each patient. In this case, the patients are divided into four subgroups (one for each level of the tree) denoted by *FLT3^Mut^*, *NRAS^Mut^*, and inv(16) biomarkers (**Table 1**; **Figure 5**).

**Table 1:** MOM Output: Patient stratification based on drug response to guide clinical decision-making.

Name	Biomarkers	Drug	Patients Treated
*Subgroup 1*	*FLT3* ^Mut^	Quizartinib	103
*Subgroup 2*	*FLT3* ^WT^ & inv(16)	Trametinib	15
*Subgroup 3*	*FLT3* ^WT^ & no inv(16) & *NRAS^Mut^*	Selumetinib	42
*Subgroup 4*	FLT3^WT^ & no inv(16) & *NRAS* ^WT^	Crizotinib	159

Following the new therapeutic strategy, the first biomarker is *FLT3^Mut^*-including *FLT3-ITD*. Patients carrying *FLT3^Mut^* would be treated with Quizartinib a 2^nd^ generation *FLT3* inhibitor that is currently facing several clinical trials showing an increase in overall survival for AML patients (18). This group of patients represents 30% of patients (25), in our study, 103 patients out of 319 belong to this group. The second subgroup comprises 15 patients and is characterized by *FLT3^WT^* and the inv(16), which generates the fusion gene *CBFβ-MYH11*. Patients with these biomarkers are sensitive to Trametinib, a *MAPK* inhibitor that prevents cell replication and has been initiated in phase I clinical trials for hematological malignancies (30). Interestingly, within this group, the patients with *NRAS^Mut^* (4 out of 16) are the most sensitive to Trametinib. The third group is defined by the absence of previous biomarkers and *NRAS^Mut^*. This subgroup poses special interest in the research as *NRAS* is one of the biomarkers most closely related to the general resistance to treatments of this disease (31). *NRAS* gene variants are mutually exclusive with *FLT3* variants (p-value<0.05; **Supplementary Figure 16**). Patients within this subgroup are sensitive to Selumetinib, a *MAPK* inhibitor that has started clinical trials for acute lymphoblastic leukemia in the UK (32), it is a mitogen-activated pathway inhibitor, which could inhibit *RAS* pathway functionality (33).

Finally, the fourth subgroup comprises the rest of the patients with none of the above mutational biomarkers but with other possible mutated biomarkers, for which the best treatment is Crizotinib -an *ALK* and *MAPK* inhibitor- approved by the FDA for lung cancer. It has not been enrolled in clinical trials for AML. Nevertheless, it has been used in studies of high-risk AML patients, with *TP53^Mut^* and obtained very promising results (34).

To validate the MOM’s algorithm, we first run MOM on the BeatAML *ex-vivo* dataset using 10-fold cross-validation and compare the results that MOM outputs with each fold. This analysis shows that the MILP optimization returns robust results as 90% folds share 4 out of 5 biomarkers (**Supplementary Figure 5**). Specifically, *FLT3^Mut^* and *NRAS^Mut^* subgroups appear in 10 out of 10 folds and subgroup with inv(16) in 3 out of 10 folds.

We then evaluated the treatment guideline proposed by running MOM with BeatAML within three independent AML datasets: two large-scale loss-of-functionality experiments that used both RNAi (DEMETER 2 (35)) and *CRISPR*-Cas9 (CERES (36, 37)), and an additional large-scale cell-drug sensitivity analysis (Genomics of Drug Sensitivity in Cancer, GDSC (1, 38, 39)). We characterize cell lines using the Cancer Cell Line Encyclopedia’s (CCLE (40, 41)) genetic variant files, from which we clustered the AML cell lines into the four subgroups predicted by MOM using as input BeatAML. For CERES and DEMETER 2, we identified the main target and model drug effects to be proportional to the depletion of their target, which is the information these databases included.

For each subgroup, we compared each experiment’s sensitivity (CERES score, DEMETER 2 score, and GDSC-IC50) dividing patients according to the presence of the biomarkers predicted by MOM in BeatAML and summing their sensitivity scores of the other three databases. We compute the sensitivity scores for the 4 subgroups, and the 3 datasets independently DEMETER2 (n=18 AML cell lines), CERES (n=14 AML cell lines), and GDSC (n=23 AML cell lines) (**Figure 5**). For the GDSC dataset, we compared the IC_50_ value from the cell lines with the selected biomarker and without the biomarker for a given subgroup drug. Finally, we performed an additional validation using DEMETER RNAi dataset (n=15 AML cell lines; **Supplementary Figures 7-8**).

The change in sensitivity for the selected treatments is strongly significant using the MOM’s predicted biomarkers in the three experiments (p-values of 5.5e-05, 6.8e-06, and 5.5e-04 for CERES, DEMETER2, and GDSC, respectively; **Supplementary Figures 9-11**). Remarkably, inv(16) is difficult to be validated using cell lines, as commercial cell lines mostly lack this alteration. The ME-1 cell line is an exception to that, but GDSC is the only dataset that includes the translocation. Although this comparison is not statistically significant due to the lack of data, the GDSC-IC50 of ME-1 is 30 times lower than the average of cells without inv(16).

We carried out a functional enrichment analysis to unveil the patient genotype according to the stratification proposed by MOM. We calculated the differentially expressed genes that are representative of each subgroup (**Supplementary Tables 2-5**) and computed the enriched biological functions of patients that belong to each group. The first subgroup, defined by *FLT3^Mut^*, is characterized by downregulation in Myeloid Leukocyte Migration (adjusted p-value< 5e-3; **Supplementary Figure 23**, **Supplementary Table 7**), this result is present in other functional enrichment studies involving *FLT3* mutated subgroup (42, 43). This subgroup has been repeatedly mentioned in literature and *FLT3* inhibitors are being implemented in the clinic (18). The second subgroup, defined by samples with inv(16) and *FLT3^WT^* shows upregulated cell proliferation (adjusted p-value< 1e-3) including angiogenesis and endothelial cell migration upregulated among others (**Supplementary Figure 24**, **Supplementary Table 8**), also described in other studies concerning this genetic aberration (44–46).

We also found that the *NRAS^Mut^* subgroup is related to the downregulation of alternative splicing (AS; adjusted p-value< 0.2; **Supplementary Figure 27**, **Supplementary Table 11**). This subgroup has an upregulation of the transforming growth factor-beta (TGF-β) signaling pathway (adjusted p-value< 5e-03; **Supplementary Figure 26**, **Supplementary Table 10**), which is mentioned in other studies concerning AS, especially in myelodysplastic syndromes (47, 48). Furthermore, several studies have attempted to address the relationship between AML and AS, with promising results (49–51).

Finally, patients who do not have the previous biomarkers, have a downregulation in the amino acid catabolism process (adjusted p-value< 0.05; **Supplementary Figure 29**, **Supplementary Table 13**), i.e. they are less able to metabolize amino acids than the rest of the subgroups (52). A study demonstrates that for a subpopulation of AML leukemia stem cells the metabolism of amino acids from the medium is essential, and its absence leads to cell death (52). Further description of the enriched functions for each subgroup, as well as their relationships and statistical significance, can be found in the supplementary material (**Supplementary Figures 22-29**, **Supplementary Tables 6-13**).

## 3 Discussion

Despite the advances in drug *ex-vivo* screening and computational methods for precision medicine, there are technical issues that limit their translation to clinical practice. Some of these issues are the influence of drug toxicity, the enormous number of statistical hypotheses, the complexity of developing algorithms understandable by the clinician, and the difficulty of proposing an effective treatment guideline that assigns the best drug for each patient. MOM faces and solves each of these challenges.

These statements are not yet covered by current AI strategies, which are focused on increasing accuracy and sensitivity regardless of the complexity of the end model (7, 53). In these AI methods, the absence of interpretability of the feature used for classification prevents further research and downplays the need for clinically defined subgroups (54–56). Indeed, the need of developing XAI algorithms is not only related to easing the diagnosis pipeline in cancer but also to increase and facilitate that the pharma industry brings new drugs and biomarkers to market. Drug regulators -such as the Food and Drug Administration- value that the process to unveil novel biomarkers is robust and transparent (10). In contrast, the patient stratification guideline provided by MOM has the following characteristics, i) allows treatment assignment by using a simple genetic panel, ii) the results are non-stochastic, they are the same for all possible re-runs of the model, iii) the algorithm outputs a decision tree for treatment guidance.

IC_50_, EC_50,_ and AUC (used for example in (1, 6, 38)) are reasonable metrics to determine the efficacy of a drug. None of them, however, considers the overall toxicity of the drug. Using IC50* in the optimization problem, we focus on the differential effectiveness of a drug among different patients, and therefore, drugs that are toxic for most samples will not be included in the solution.

IHW provides us with the ability to increase the power of tests and reduce the FDR. With this strategy, we are also able to identify the direction of the influence of genetic events in drug response, i.e., whether it defines sensitivity or resistance. With this approach, we successfully detected *FLT3* as highly influential in terms of sensitivity to treatment, which is coherent with other studies (25). *NRAS*, instead, showed as a mutation associated with treatment resistance also coherent with literature (26, 31). One promising conclusion for this study is that we managed to find a drug for which *NRAS* correlates to drug sensitivity.

XAI defined by MILP ensures that the subgroups obtained are optimal. This feature is not common to other classification methods. However, it also presents two main limitations. The first one is computational resources, which increases exponentially with the number of possible biomarkers, drugs, or patients (on a standard desktop, the presented work required 2.5 hours of computing time). In addition, the incorporation of new non-binary diagnostic markers requires the redefinition of the model. However, once the optimization problem is solved, assigning a treatment to a novel patient is immediate.

Our AML patient stratification includes a subgroup defined by the absence of a genetic mutation, i.e., wild type. It also includes patients who have *TP53^Mut^* genotype, which are biomarkers associated with poor prognosis (14). MOM recommends treating these patients with Crizotinib, a drug used in other studies with *TP53^Mut^* AML patients which in fact showed very promising results (34). In addition, this subgroup shows a deficiency in amino acid metabolism which may lead to alternative treatment therapies based on metabolomics.

The subgroup defined by the *CBFβ-MYH11* fusion gene appears characterized in a very small percentage of AML cell line cohorts but is nevertheless present in 7% of AML patients (57), which enhances the relevance of this biomarker. *CBFβ-MYH11* is a clear indicator of sensitivity to Trametinib, a clinical drug that inhibits cell replication pathway (58), which, in turn, appeared as an upregulated biological process in this subgroup. In the remaining subgroups, *FLT3^Mut^* is widely described in the literature (25). In contrast, *NRAS^Mut^* appears as a biomarker of sensitivity for Selumetinib and has downregulated the alternative splicing (AS) process. This subgroup contains, on balance, effective treatment for a resistance-associated mutation and a new line of research linking alternative splicing and AML.

It is remarkable the appearance of three different *MAPK* inhibitors in the proposed therapeutic strategy, which is coherent with the disease behavior. Our biomarker analysis revealed that the RTK-RAS pathway is the most affected in our cohort of AML samples (**Supplementary Figures 18-19**). Of all drugs suggested as treatment, only Quizartinib is clinically approved for AML patients (15). This study aims to accelerate -once the results are validated in cell lines and murine models- the process of approving these drugs for AML.

The validation of the results is challenging in a real cohort since most patients are treated with standard induction cytotoxic therapy (only 7.5% of AML patients in TCGA are treated with other treatments). We propose a strategy to take advantage of cell lines loss-of-function datasets. Nevertheless, even using cell lines -that are quite different from ex vivo samples- we validated the subgroups and the IC_50_ of the lines with indication was significantly better than the IC_50_ of those without indication. Therefore, in the absence of clinical data for validation, we consider the results using cell lines data to sufficiently support this study.

The concept of MOM is also applicable to other disease types using *ex-vivo* experiments as well as to other sensitivity measurements, leaving an open door for new patient stratifications based either on drug response or even on any other experiment to measure the effectiveness of certain drugs in the future. We believe that XAI will help doctors and regulators understand AI medical decisions and, therefore, ease the translations of AI analysis of drug screening experiments to clinical practice.

## 4 Methods

### 4.1 Filter and normalization

#### 4.1.1 Filtering and imputation

We used data from *ex-vivo* experiments, WES, and RNA-Seq from 319 Acute Myeloid Leukemia (AML) patients included in the BeatAML cohort (19). Data was filtered to ensure all samples contained the gene variants and drug sensitivity information, the new dataset containing genomic aberrations and drug IC_50_ for the same patients was used as a starting point for the study. Genetic variant samples were previously pathogenically filtered by Tyner et al. (19) and we defined as a biomarker a genetic variant present in more than 1% of the patients (n≥4), leaving a total number of 64 possible biomarkers.

For missing drug sensitivity information in the ex-vivo experiments, we imputed the missing data using the *k*-Nearest Neighbourhood (kNN) Impute method, from Impute R package (59) (version 1.68.0). An analysis of the missing values −both patients and drugs− is included in the supplementary material

#### 4.1.2 Drug normalization: from IC_50_ to IC50*

Initially, we tried to use as drug sensitivity values the half-minimal inhibitory concentration, (IC_50_) i.e., the concentration of a drug -in micro molar- for which half of the cell from the ex-vivo experiment die. Instead of using the IC_50_, we propose the usage of an incremental version of the IC_50_, named IC50*. As described in the results section, the usage of IC50* instead of IC_50_ is a convenient way to deal with the different toxicity of the drugs under study

After imputation, IC_50_ values were taken the log_10_ logarithm, normalized by subtracting the IC_50_ mean value for each drug, and these scores were made negative by subtracting an offset to the normalized IC_50_ value –the optimization model assumes negative values of drug sensitivity. The obtained drug sensitivity values are named IC50*. The transformation from IC_50_ to IC50* is represented in equation (1). Despite the formidable aspect of the formula, IC50* is simply an incremental and version of the logarithm of IC_50_ with an offset.

Let IC_50_ be a *T x P* matrix, with *T* the total number of drugs and *P* the total number of patients, for which each element ic50_t,p_ is a value contained in (0,10] µM.


(1) $$ic50_{t,p}^{*}=(\log_{10}(ic50_{t,p})-1)-\frac{1}{P}\sum_{p=1}^{P}\left(\log_{10}(ic50_{t,p}\right)-1)-\mathrm{-max}\left(\left(\log_{10}(ic50_{t,p})-1)-\frac{1}{P}\sum_{p=1}^{P}\left(\log_{10}(ic50_{t,p}\right)-1\right)\right)$$

The obtained IC50* is a *T x P* matrix containing the new drug sensitivity values.

### 4.2 Drug-biomarker association

Following with MOM’s second step, we implemented a two-tailed Wilcoxon test to assess whether a biomarker influences the sensitivity of each the treatment. Each biomarker is tested against each drug and these associations were ranked according to the p-value. The p-values were adjusted following the methodology described by Gimeno *et al.* (22), using the R package *IHW* (21) (version 1.22.0). The package provides (given the p-values and the covariates –in our study genetic alterations–) a weight for each covariate related to its influence on the p-value significance.

Using these results, we included two consecutive filters. Firstly, we selected the biomarkers whose relative importance (the weight outputted by IHW) is larger than zero. IHW assigns a strictly positive weight to biomarkers relevantly correlated to the potency of a drug. Afterwards, we removed the drugs with no statistically significant relationship to the selected biomarkers (IHW p-value >0.05).

After this analysis, 122 treatments (biomarker-drug associations), with ΔIC50*>0.2 (including *vs* lacking the biomarker) and adjusted p-value<0.05 were considered for therapy.

### 4.3 MOM: MILP Module

Finally, in the third step, we proceed with the treatment assignation. We developed a MILP module described in the Results section. This module receives as input the 122 treatments and solves an optimization problem (described in detail in de **Supplementary Material**) MILP results can be directly translated into a decision tree for guiding clinical decision-making. The number of levels of the tree was set to four. Each level of this tree will be defined as one therapeutic AML subgroup and each subgroup is defined by a biomarker and a recommended drug.

Additional information regarding the algorithm, its *in-silico* validation, and its performance can be found in **Supplementary Material** (Section **Supplementary Methods**).

### 4.4 External cohort validation

For validating the different subgroups, we compared patients that are given a drug in a specific subgroup against the remaining non-treated patients. We validated our results using cell lines, specifically, used 2 different large-scale gene essentiality experiments including RNAi (DEMETER 2 (35)) and CRISPR-Cas9 (CERES (36, 37)), and an additional large-scale cell-drug sensitivity analysis (Genomics of Drug Sensitivity in Cancer, GDSC (1, 38, 39)). We characterized the cell lines using the Cancer Cell Line Encyclopedia (CCLE (40, 41)) genetic variants files, from which we were able to divide the cells into different subgroups.

We performed the following test for validation. Cells were divided into two groups. The first group includes cells with the biomarker associated to that subgroup, and the other group, contains the cells without the biomarker that had not been previously treated. This comparison was computed for the 4 subgroups, and the 2 datasets DEMETER 2, and CERES. DEMETER 2 and CERES were compared using the viability score that corresponds to knocking out the corresponding targets for each drug. For the GDSC dataset, we used the IC_50_ value provided in the experiments. All tests were one-tailed Wilcoxon’s test to check that the sensitivity increase in the cells with the biomarker.

### 4.5 Functional analysis of the subgroups

Functional analysis of the subgroups was performed using gene expression data from the BeatAML (19) cohort. We performed a differential gene expression analysis using limma R package (60) (version 3.50.3). The contrast matrix compared one group against all the others, therefore, there was a different contrast for each group.

Genes differentially expressed were ranked according to its t-statistic, if t >0, genes were considered overexpressed, if t<0, genes were considered underexpressed. For each subgroup, we selected the top 500 over and under expressed genes and performed a Gene Ontology Enrichment Analysis (GEA) using Fisher’s Test. We analyzed the biological process ontology. Enriched functions on the overexpressed genes were upregulated, and functions obtained from the underexpressed genes were considered to be downregulated. The statistics were computed using clusterProfiler R package (61) (version 3.10.1). We set an adjusted p-value cutoff of 0.2 for considering a function differentially enriched, adjusted p-values were computed using the Benjamini-Hochberg procedure.

## Data availability statement

Publicly available datasets were analyzed in this study. This data can be found here: http://vizome.org/additional_figures_BeatAML.html

## Author contributions

MG, AR and FC conceived this study. MG, EJ-E, SV, XA, FP, AR and FC designed the MOM requirements and provided biological insights for the assignation problem. MG and FC developed the pre-processing pipeline. MG, AR and FC carried out the computational implementation and validation. All authors contributed to the article and approved the submitted version.

## Funding

This research was funded by Cancer Research UK [C355/A26819] and FC AECC and AIRC under the Accelerator Award Programme, and Synlethal Project (RETOS Investigacion, Spanish Government).

## Acknowledgments

The authors would like to thank Francisco J. Planes, Iñigo Apaolaza, and Luis V. Valcárcel for the fruitful comments on the development of the methodology. The authors would like to acknowledge Katyna Sada for proof-reading and her suggestions to improve readability.

## Conflict of interest

The authors declare that the research was conducted in the absence of any commercial or financial relationships that could be construed as a potential conflict of interest.

## Publisher’s note

All claims expressed in this article are solely those of the authors and do not necessarily represent those of their affiliated organizations, or those of the publisher, the editors and the reviewers. Any product that may be evaluated in this article, or claim that may be made by its manufacturer, is not guaranteed or endorsed by the publisher.
