# Supplementary material for: Explainable artificial intelligence for precision medicine in acute myeloid leukemia
Source: Front Immunol. 2022 Sep 29;13:977358. doi: 10.3389/fimmu.2022.977358 (PMC9556772; doi:10.3389/fimmu.2022.977358)
Supplement: Supplementary file 2 [file DataSheet_2.pdf]

SUPPLEMENTARY: Explainable Artificial Intelligence for Precision Medicine in Acute Myeloid Leukemia

Marian Gimeno[1], Felipe Prosper[2,3,4], Xabier Agirre[2,3], Edurne San Jose-Eneriz[2,3], Sara Villar[4], Angel Rubio[1,5‡], and Fernando Carazo[1,5‡]

[1]Departamento de Ingeniería Biomédica y Ciencias, TECNUN, Universidad de Navarra, San Sebastián, Spain[2]Programa [2]Hemato-Oncología, Centro de Investigación Médica Aplicada, IDISNA, Universidad de Navarra, Pamplona, Spain.

[3]Centro de Investigación Biomédica en Red de Cáncer (CIBERONC), Madrid, Spain.

[4]Departamento de Hematología, Clínica Universidad de Navarra, Universidad de Navarra, Pamplona.

[5]Instituto de Ciencia de los Datos e Inteligencia Artificial (DATAI), Universidad de Navarra, 31080, Pamplona, Spain.

‡Corresponding Authors. These authors share correspondence. Correspondence and requests for materials should be addressed to A.R. (email:arubio@tecnun.es) and F.C. (email: fcarazo@tecnun.es).

Index of figures 2
Supplementary Methods 5
Missing Data Processing 5
MILP optimization model 6
Performance of MOM 11
Supplementary Results 16
Standard Classification of Patients 16
BeatAML Treatment Stages 16
Biomarker Analysis 17
Individual Drug Biomarker Associations 22
Individual Analysis of the Predicted Subgroups 25
References 34

In the supplementary material, we have included an index of figures, supplementary methods, and supplementary results. The supplementary methods include a description of the missing values in the data as well as a thorough description of the optimization procedure, and several *in-silico* validations.

On the other hand, the supplementary results include a description of the standard classification of the patients, an analysis of the biomarkers (prevalence of each of them with the aid of the maftools R package), an analysis of the known drug biomarker associations, and a profile of enriched functions of the four subgroups according to the predicted treatment.

Index of figures

***Figure S1**: **NAs distribution in IC50* data.** A) shows the percentage of NAs by patients. Patients with more than 80 % of missing data (dotted blue line) were excluded from the analysis. B) This graph shows the percentage of NAs by drug, drugs with more than 70% (dotted red line) were excluded from the analysis.* *5*

***Figure S2: Heatmap showing matrix $OM_{p,t}$.** Located in the rows are all the different individual associations called treatments, and in the columns are the patients. This matrix contains the sensitivity score for the patients having the biomarkers associated with each treatment in the row. Also, groups labels shown in the heatmap include relevant biomarker status. The score in blue represents sensitivity while in red refers to resistance or absence of the biomarker.* *8*

Figure S3: New Patient Stratification showing the score for each of the patients on each of the subgroups. *9*

***Figure S4: Heatmap of all possible treatments labelled by selected treatment**. This heatmap shows the response for each patient to all possible treatments. Treatments are contained in the rows and patients in the columns. Columns are labelled according to subgroup. The content is the sensitivity score in blue represents that the patient is sensitive to that treatment and in red that the patient is resistant to the treatment or the patient is not having the biomarker associated or it has been treated before.* *10*

***Figure S5: Results 10-fold Cross-validation.** In the Y-axis is plotted the number of folds in which each subgroup appeared. In the X-axis all the different subgroups that appeared in the cross-validation.* *11*

***Figure S6: Results Sensitivity**. We plotted the sorted ranks of the drug predicted by MOM for each patient. This plot shows that the suggested treatment was the best one in 3% of the cases, within the top 10% in 30% of the cases, within the first quartile in 46% of cases. The statistical significance for each of*

the thresholds can be stated using a Bernoulli distribution. We also included the p-values according to this distribution using thresholds for 1% (p-value=0.005), 5%(p-value=4.58e-11), 10%(p-value=2.24e-23) and 25%(p-value= 4.32e-17) 12
***Figure S7: Validation in cell lines using DEMETER Score**. In red the cell lines with the biomarker associated with the treatment; in blue the cell lines without the biomarker. On the left, $FLT3^{Mut}$ subgroup, on the right $NRAS^{Mut}$ subgroup.* 13
***Figure S8: Overall biomarker effect in the DEMETER cohort.** All cell lines with the biomarker for the different subgroups are shown in red. In blue are shown all the cell lines that were not having the associated biomarker. P.value was computed using Wilcoxon's Test. The smaller the DEMETER Score is, the more sensitive the cells are.* 13
***Figure S9: Overall biomarker effect in the DEMETER2 cohort**. All cell lines with the biomarker for the different subgroups are shown in red. In blue are shown all the cell lines that were not having the associated biomarker. P.value was computed using Wilcoxon's Test. The smaller the DEMETER2 Score is, the more sensitive the cells are.* 14
***Figure S10: Overall biomarker effect in the CERES cohort.** All cell lines with the biomarker for the different subgroups are shown in red. In blue are shown all the cell lines that do not have the associated biomarker. P-value was computed using Wilcoxon's Test. The smaller the CERES Score is, the more sensitive the cells are.* 14
***Figure S11: Overall biomarker effect in the GDSC cohort**. All cell lines with the biomarker for the different subgroups are shown in red. In blue are shown all the cell lines that were not having the associated biomarker. P.value was computed using Wilcoxon's Test. The smaller the IC_{50} is, the more sensitive the cells are.* 15
***Figure S12: Treatment Stages in the BeatAML cohort. A)** From the 319 patients contained in the study coming from the BeatAML cohort, the chemotherapy phase veries considerabily. B) Distribution of the patients accounting for the different chemotherapy phases excluding the patients with missing info or who did not take any chemotherapy.* 17
***Figure S13: Mutational Status of Beat AML cohort**. This plot shows the different types of genetic variants, the lateral barplot shows the sum of all the genetic alterations in all patients and colours the type of variant. In the horizontal axis we have the individual patients' information, showing some patients having up to 70 co-occurring mutations. Of the 608 patients, 538 had at least one genetic variant (88.16%).* 19
***Figure S14: Genetic Variant Type Summary.** Variant classification plot represents the different genetic variant types, in terms of functionality and we see that the most common is missense variant, the Variant type plot represents the type of structural variant, whether they are Single Nucleotide Variants (SNVs) or Indels with a clear predominance of SNVs, SNV classification plot shoed the type of mutational signature that is predominant with the signature C>T that is quite frequent in malignant cancer types followed by C>A which is associated with environmental exposure[10]. The Variants per Sample Plot, tells that there is a median of 8 variants per patient. The variant classification summary plot summarises all the prior plots and, finally, the Top 10 mutated genes plot, shows for each of the top 10 genes the type of mutation.* 19
***Figure S15: Transitions(Ti) and Transversions (Tv) landscape in Beat AML cohort**. Ti vs. Tv plot shows the number of the transitions and transversions showing that even when transversions seem most probable to occur, transitions are more present in this cohort. Boxplot showing overall distribution of six different conversions and as a stacked barplot showing the fraction of conversions in each sample. The most common transition is C>T, followed by a transversion of C>A.* 20
***Figure S16: Somatic Interactions.** Mutually exclusive or co-occurring set of genes calculated using pair-wise Fisher's Exact test. Associations plotted in green represent Co-occurrence while brown is a sign of mutual exclusivity. Stars are assigned to associations with P< 0.05. We appreciated that FLT3 and NPM1 variants are co-occurrent, and FLT3 and TP53 and NRAS and IDH2 are mutually exclusive respectively.* 20
***Figure S17: Translocations and SNVs**. All translocations can be identified by a gene fusion product: inv(16) with CBFβ-MYH11, inv(3) with RPN1-EVI1, t(9;11) with MLLT3-MLL, and t(8;21) with RUNX1-RUNX1T1. From these translocations, inv(16) appears in co-occurrence with FLT3, KIF20B, and ADAMTS7 variants. Whereas t(9;11) can appear with NRAS variants and inv(3) with KIT variants* 21

Figure S18: Oncogenic Signalling Pathways altered in Beat AML cohort. *The barplot on the left represents the proportion of genes that are altered in the pathway, whereas the barplot on the right, shows the proportion of samples that are having an alteration in that pathways.* _________________ 21
Figure S19: RTK-RAS pathway alterations*. In the Y-axis all genes are included in the pathway, in blue the oncogenes and red tumour suppressor genes. In the X-axis all the samples with RTK-RAS altered and the red marks show the pathway genes altered for each sample.* ___________________________________ 22
Figure S20: Beat AML Drugs distribution. *Drugs studied in the BeatAML cohort cover a wide variety of different cancers and diseases, from which 24% are designed, experimental, or already prescribed for AML, 16% for other leukemias, 10% are shown concerning multiple myeloma, and 4% for different types of lymphomas. This means that 54% of the drugs have been studied for hematological malignancies.* _ 23
Figure S21. Relevant Individual Drug-Biomarker Associations. *In blue the patients with the biomarker in orange the patients without the biomarker. P-value association is corrected according to the main manuscript. The score shows the differential IC_{50}, more negative means more sensitive.* ____________ 24
Figure S22: FLT3^{Mut} – Quizartinib Upregulated GO enrichment Functions ________________________ 26
Figure S24: FLT3^{WT} & Inv(16) – Trametinib GO upregulated enrichment Functions ________________ 28
Figure S25: FLT3^{WT} & Inv(16) – Trametinib GO downregulated enrichment Functions _____________ 29
Figure S26: FLT3^{WT} & no Inv(16) & NRAS^{Mut} – Selumetinib GO upregulated enrichment Functions ___ 30
Figure S27: FLT3^{WT} & no Inv(16) & NRAS^{Mut} – Selumetinib GO downregulated enrichment Functions _ 31
Figure S28: Rest – Crizotinib GO upregulated enrichment Functions ____________________________ 32
Figure S29: Rest – Crizotinib GO downregulated enrichment Functions __________________________ 33

Supplementary Methods

Missing Data Processing

We used KNN Impute[1] to impute the missing values. No values were imputed for patients with NA % higher than 80% and drugs whose proportion of missing values was above 70%. These thresholds seem to be high, however, the distribution of the NAs shows that the vast majority of either the patients or the drugs have less than 20 % of missing values (**Figure S1**). Since the proportion of missing values was reasonably low we did not compare different algorithms to impute them. We used KNNImpute since it has shown to be reliable and widely used.

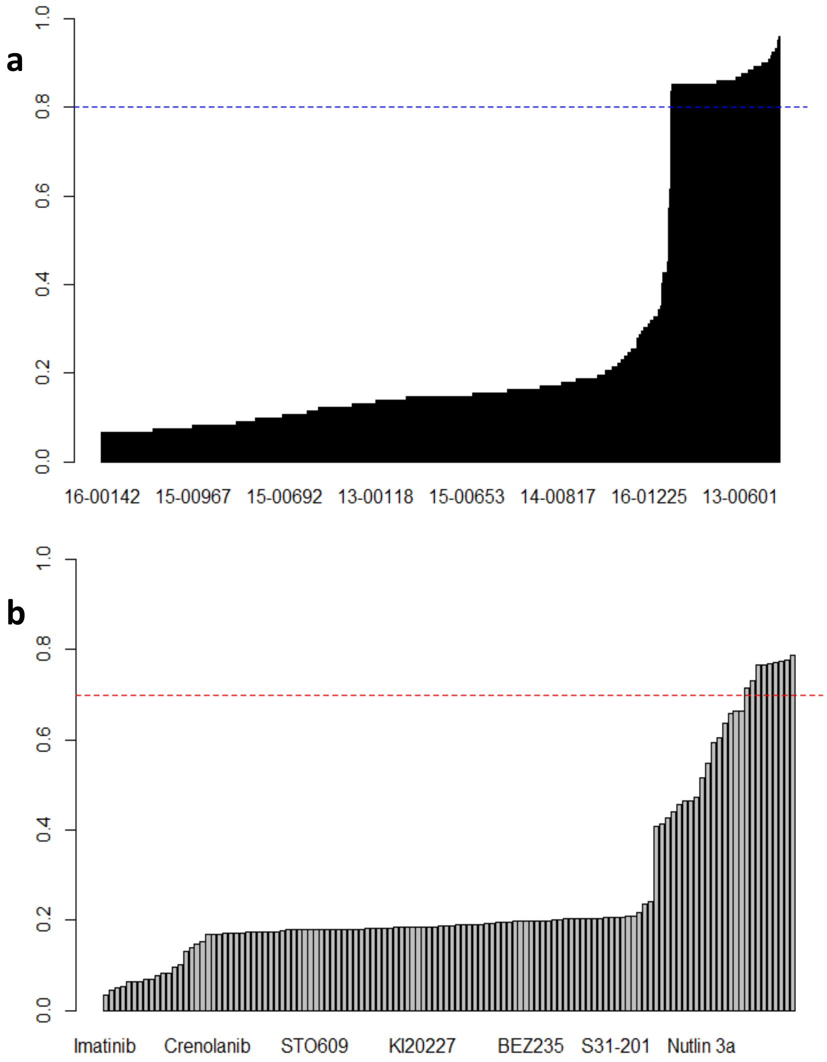

Figure S1: **NAs distribution in IC50* data.** A) shows the percentage of NAs by patients. Patients with more than 80 % of missing data (dotted blue line) were excluded from the analysis. B) This graph shows the percentage of NAs by drug, drugs with more than 70% (dotted red line) were excluded from the analysis.

MILP optimization model

The core of MOM is an integer programming optimization model that predicts the combination of drugs and biomarkers that optimize patient response to treatment (i.e., **IC50***). Let us define a treatment as a combination of a drug and a companion biomarker. The solution to the optimization problem consists of a set of treatments that will be applied sequentially to patients in a defined number of steps (one treatment per step).

Let *S, P,* and *T* be the total number of possible steps, patients, and treatments included in the study respectively. Let **OM** be an input $P\,x\,T$ matrix of essentiality, whose elements $om_{p,t}$ contains the normalized sensitivity score (IC50*) of *ex-vivo* experiments for a patient *p* and a treatment *t*, which fulfills $om_{p,t} \leq 0$. IC50* values are all negative.

Let **K** be a $P\,x\,T$ a binary matrix whose element k_{pt} denotes whether a patient *p* is eligible for the treatment *t*, i.e., the treatment's companion biomarker is present in the patient, as follows:

$$k_{pt}\begin{cases}1, & if\ the\ patient\ p\ has\ the\ biomarker\ associated\ with\ treatment\ t\\ 0, & otherwise\end{cases}$$

Let **X** be a binary $S\,x\,P\,x\,T$ array whose element x_{spt} states whether a patient *p* is treated with treatment *t* in step *s*, as follows:

$$x_{spt}\begin{cases}1, & if\ patient\ p\ is\ given\ the\ treatment\ t\ in\ step\ s\\ 0, & otherwise\end{cases}$$

Let **Y** be a $S\,x\,T$ binary matrix whose element y_{st} represents whether a treatment *t* is used in step *s*, as follows:

$$y_{st}\begin{cases}1, & if\ the\ treatment\ t\ is\ used\ in\ step\ s\\ 0, & otherwise\end{cases}$$

Given these variables, the MOM algorithm was built as a MILP optimization problem defined by the following equations.

(1) $$\text{minimize} \sum_{s=1}^{S}\sum_{t=1}^{T}\sum_{p=1}^{P} om_{pt}\cdot x_{spt}$$

(2) $$s.t. \quad \sum_{t=1}^{T} y_{st} = 1 \qquad s = 1,\dots,S$$

(3) $$\sum_{s=1}^{S}\sum_{t=1}^{T} x_{spt} \leq 1 \qquad p = 1,\dots,P$$

(4) $$x_{spt} \leq y_{st}\cdot k_{pt} \qquad s = 1,\dots,S; t = 1,\dots,T; p = 1,\dots,P$$

(5) $$x_{spt} + \sum_{n=1}^{s-1}\sum_{m=1}^{T} x_{npm} \geq\ y_{st}\cdot k_{pt} \qquad s = 1,\dots,S; t = 1,\dots,T; p = 1,\dots,P$$

The objective function of the MILP problem, **equation (1)**, is to minimize drug sensitivity scores (**IC50***) for all patients. The sensitivity score will be considered if it is included in the problem solution (x_{spt}). As drug sensitivity scores are negative, the MILP solution will intrinsically maximize the number of treated patients, as each included patient adds a negative term to the objective solution.

The proposed MILP problem has four sets of restrictions, namely **equation (2)** to **equation (5)**. **Equation (2)** is a set of *S* restrictions stating that each step consists of one treatment. **Equation (3)** is a set of *P* restrictions stating that at most one treatment must be used to treat each patient. **Equation (4)** is a set of $S\ x\ T\ x\ P$ restrictions stating that the treatment t can be applied to patient *p* in step *s*, only if (i) the patient *p* is eligible for treatment *t* based on his/her biomarkers ($k_{pt} = 1$), and (ii) the treatment t is used in step s ($y_{st} = 1$). Finally, **equation (5)** is set of $S\ x\ T\ x\ P$ restrictions that impose that the treatments included in the solution must be selected hierarchically, i.e., if we have a patient that would be eligible for two treatments, only the first treatment must be considered in the optimal solution.

To solve the model, we used CPLEX™© 12.10.0, Python 3.7.3, and the reticulate R package[2] (version 1.25.0).

The optimization model needs several input variables. The most crucial input variable is the matrix containing all the pre-processed essentiality information regarding each of the individual associations, called **IC50***$_{p,t}$. **Figure S2** contains a pre-visualization of this $\mathbf{OM}$ matrix, located in the rows all the different individual associations called treatments; and in the columns the patients. This matrix contains the sensitivity score for the patients having the biomarkers associated with each treatment in the row. Also, groups labels shown in the heatmap include relevant biomarker status. The score in blue represents sensitivity while in red refers to resistance or absence of the biomarker.

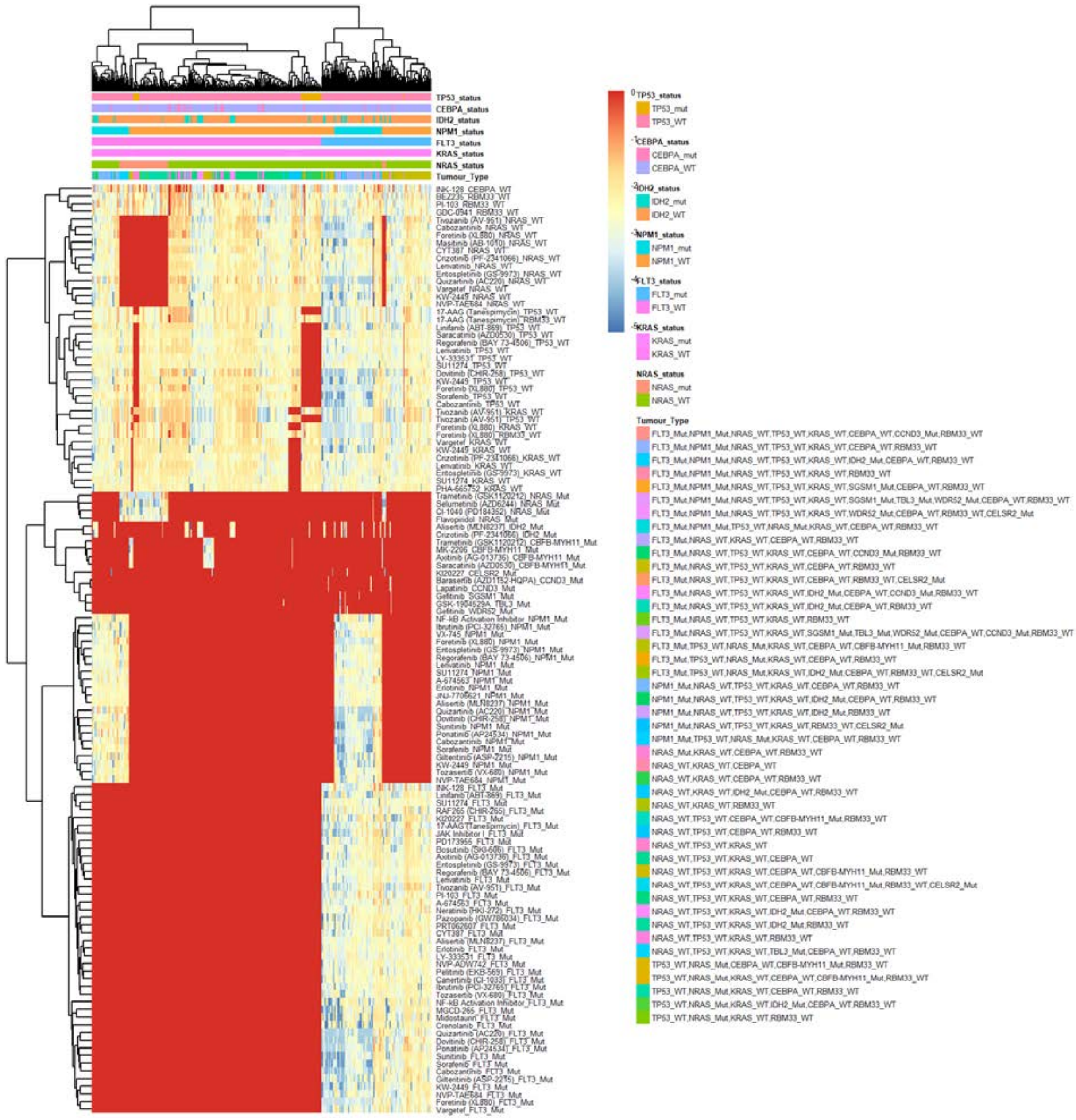

Figure S2: Heatmap showing matrix $OM_{p,t}$. Located in the rows are all the different individual associations called treatments, and in the columns are the patients. This matrix contains the sensitivity score for the patients having the biomarkers associated with each treatment in the row. Also, groups labels shown in the heatmap include relevant biomarker status. The score in blue represents sensitivity while in red refers to resistance or absence of the biomarker.

From all the possible treatments, MOM returned the 4-group patient stratification. The score of each of the subgroups compared to one another is plotted in **Figure S3**. Each of the dots represents one patient, what we can see is that within the patients treated in the last subgroup there are fewer responders, and subgroup *FLT3*^{Mut} and *NRAS*^{Mut} have more responders. **Figure S4**, shows all treatments for the patients clustered by subgroups.

To validate the algorithm we performed 10-fold cross-validation (**Figure S5**), for which subgroups *FLT3*^{Mut}-Quizartinib and *NRAS*^{Mut}-Selumetinib appeared in every fold, *NPM1*^{Mut}-KW-2449 in 3 folds, inv(16)-Trametinib in 2 folds, and *PTPN11*^{Mut}-Trametinib in 2 folds.

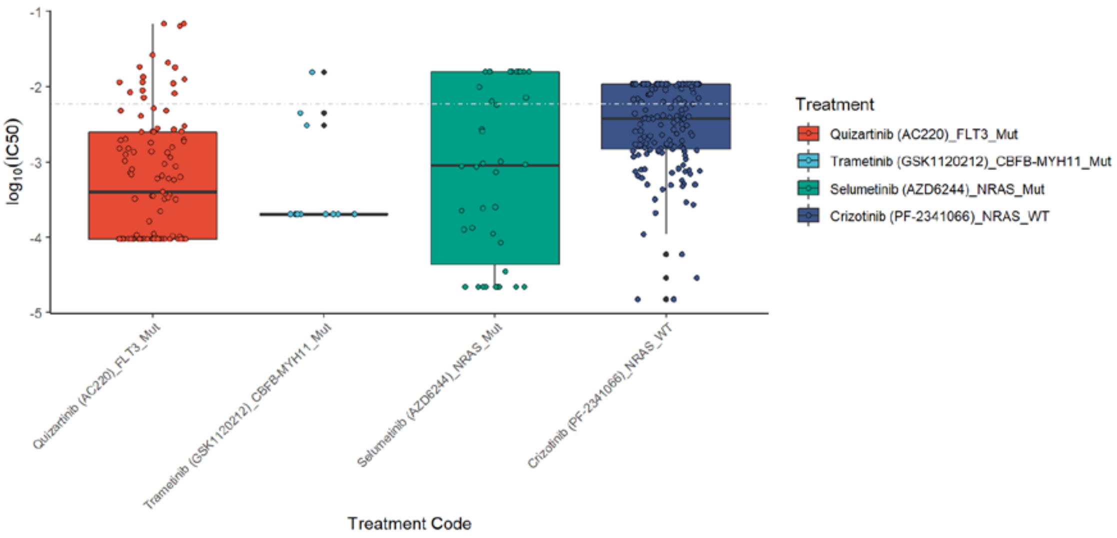

Figure S3: New Patient Stratification showing the score for each of the patients on each of the subgroups.

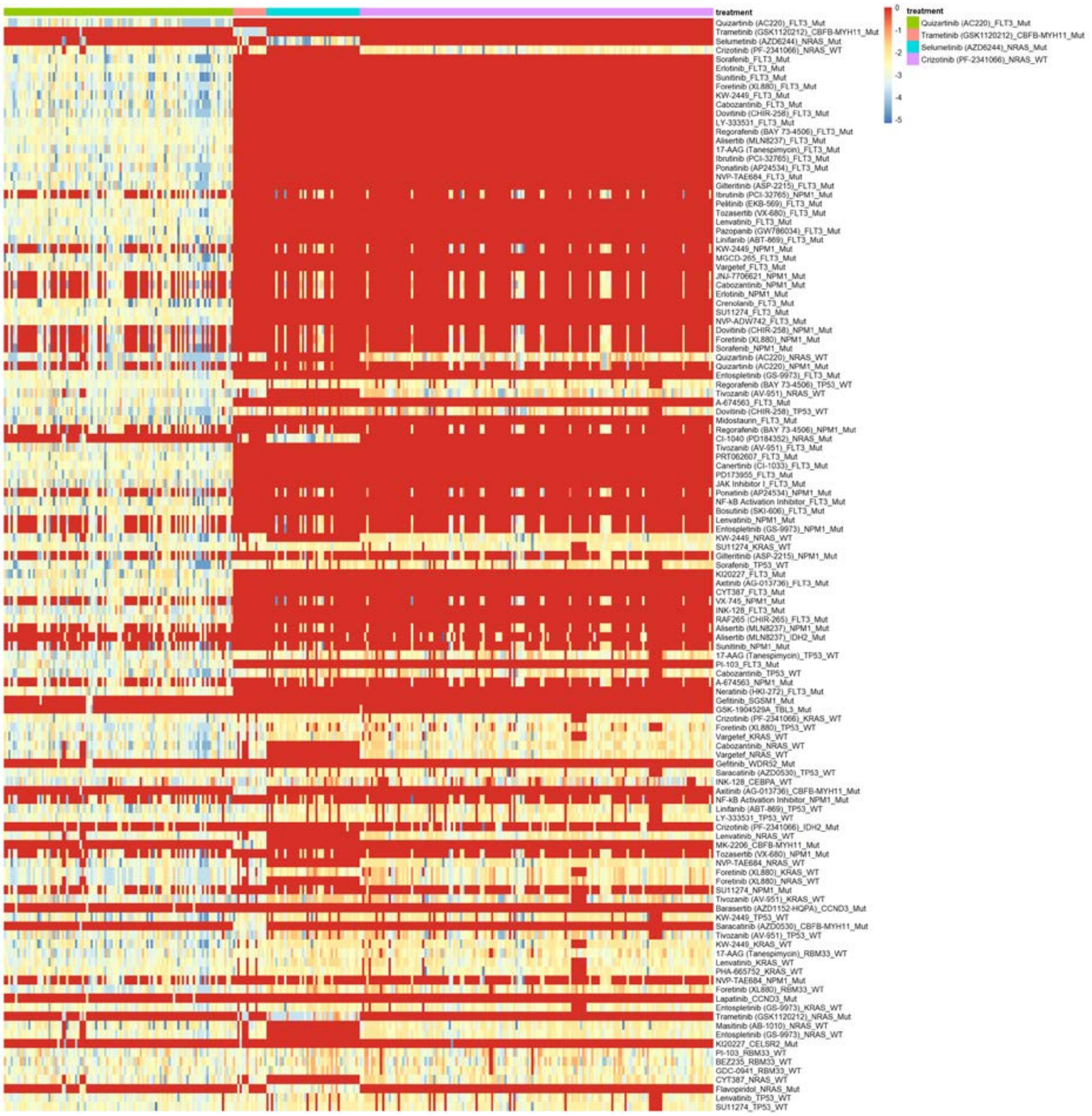

Figure S4: Heatmap of all possible treatments labelled by selected treatment. This heatmap shows the response for each patient to all possible treatments. Treatments are contained in the rows and patients in the columns. Columns are labelled according to subgroup. The content is the sensitivity score in blue represents that the patient is sensitive to that treatment and in red that the patient is resistant to the treatment or the patient is not having the biomarker associated or it has been treated before.

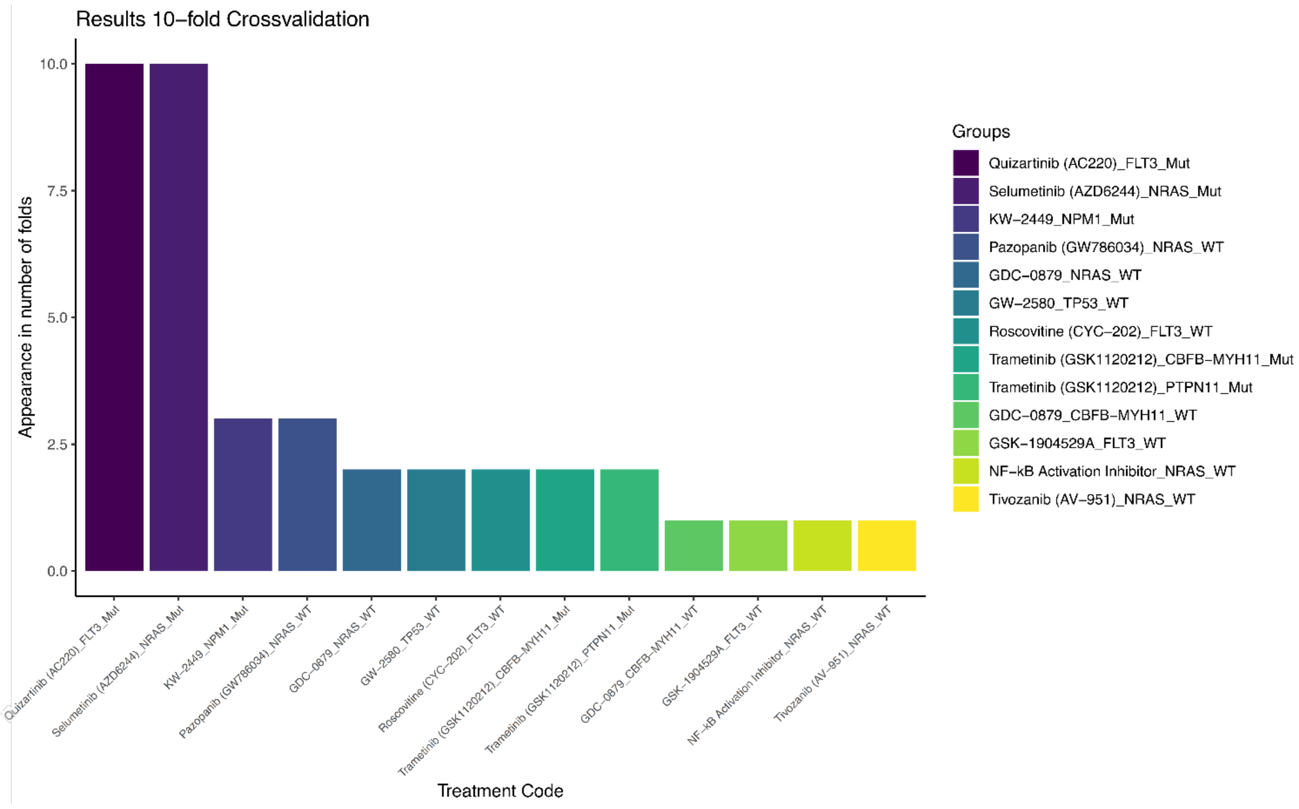

Figure S5: Results 10-fold Cross-validation. In the Y-axis is plotted the number of folds in which each subgroup appeared. In the X-axis all the different subgroups that appeared in the cross-validation.

Performance of MOM

The application of typical performance measures of machine learning (specificity, accuracy, sensitivity, ROC and PR curves, etc.) to this specific problem is not straightforward. Since MOM suggests a single drug for each patient, the potential contingency matrix will be very unbalanced: for each patient, only the drug suggested by MOM is a positive and all the other treatments are negatives. If the suggested drug is the one with the lowest IC50*, the prediction will be a true positive. Otherwise, it will be a false positive. On the other hand, all the drugs that were not selected are true negatives (except the one with the lowest IC50*). The drug with the lowest IC50*, if not selected by MOM, will be a false negative. This approach to construct the contingency matrix has several drawbacks. First, the classes are strongly unbalanced -for each patient there is only one positive and hundreds of negatives for our data. Furthermore, this approach makes no difference between predicting the second-best drug or predicting the worst drug.

Instead of this approach, we plotted (**Figure S6**) the sorted ranks of the drug predicted by MOM for each patient. This plot shows that the suggested treatment was the best one in 2% of the cases, within the top 10% in 30% of the cases and so on. The statistical significance for each of the thresholds can be stated using a Bernoulli distribution. We computed the p-values according to this distribution using thresholds for 1%, 5%, 10% (0.005, 4.58e-11, and 2.24e-23 respectively). A “prediction” algorithm that prescribes a drug by chance would show a curve close to the diagonal in this graph.

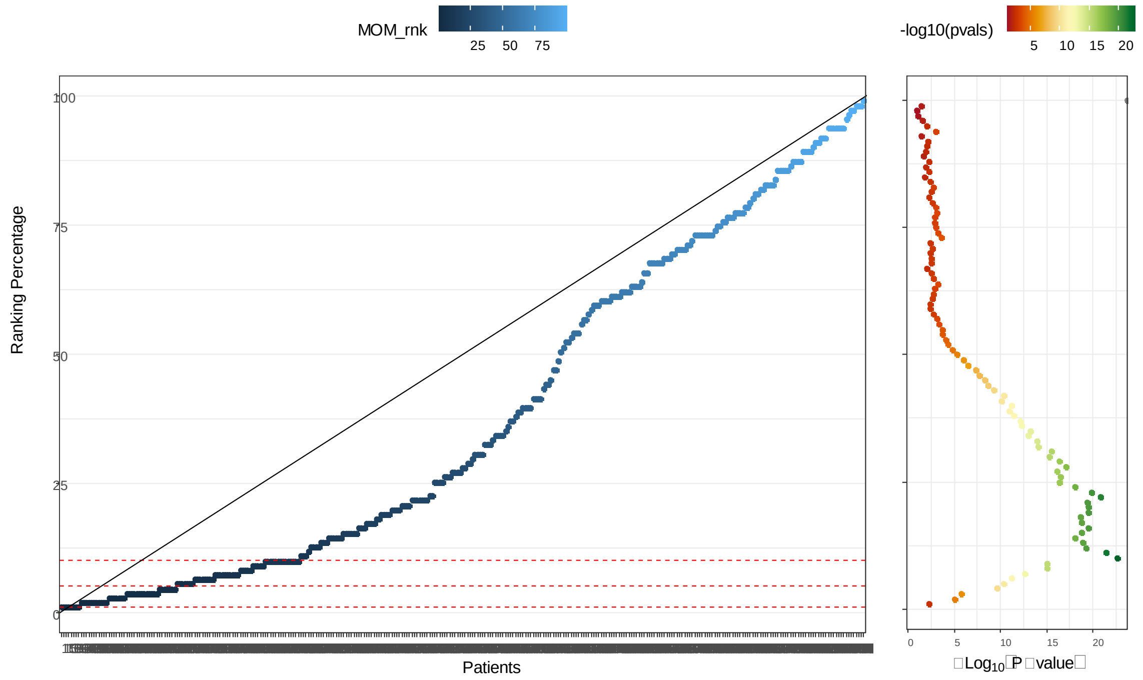

Figure S6: Results Sensitivity. We plotted the sorted ranks of the drug predicted by MOM for each patient. This plot shows that the suggested treatment was the best one in 3% of the cases, within the top 10% in 30% of the cases, within the first quartile in 46% of cases. The statistical significance for each of the thresholds can be stated using a Bernoulli distribution. We also included the p-values according to this distribution using thresholds for 1% (p-value=0.005), 5%(p-value=4.58e-11), 10%(p-value=2.24e-23) and 25%(p-value= 4.32e-17)

Moreover, we needed to validate the patient stratification in a different cohort. Due to the absence of other cohorts of ex-vivo experiments with complete clinical data, we validated the proposed stratification using cell lines. We selected 3 different types of experiments RNAi (DEMETER[3], DEMETER2[3]) *CRISPR*-Cas9 (CERES[4]) and cell lines sensitivity screening (GDSC[5]). GDSC was the only cohort to have the inv(16) biomarker. Most relevant results are included in the main manuscript. No DEMETER cell lines included the inv(16) biomarker leading to a 2-group validation. Both groups were more sensitive to the drug if carrying the biomarker (**Figure S7**). P-values were calculated using Wilcoxon's exact test. In addition, if considering only the biomarker effect, all cohorts were more significantly sensitive to the drug if their cells were carrying the associated biomarker(**Figure S8**, **Figure S9**, **Figure S10**, **Figure S11**).

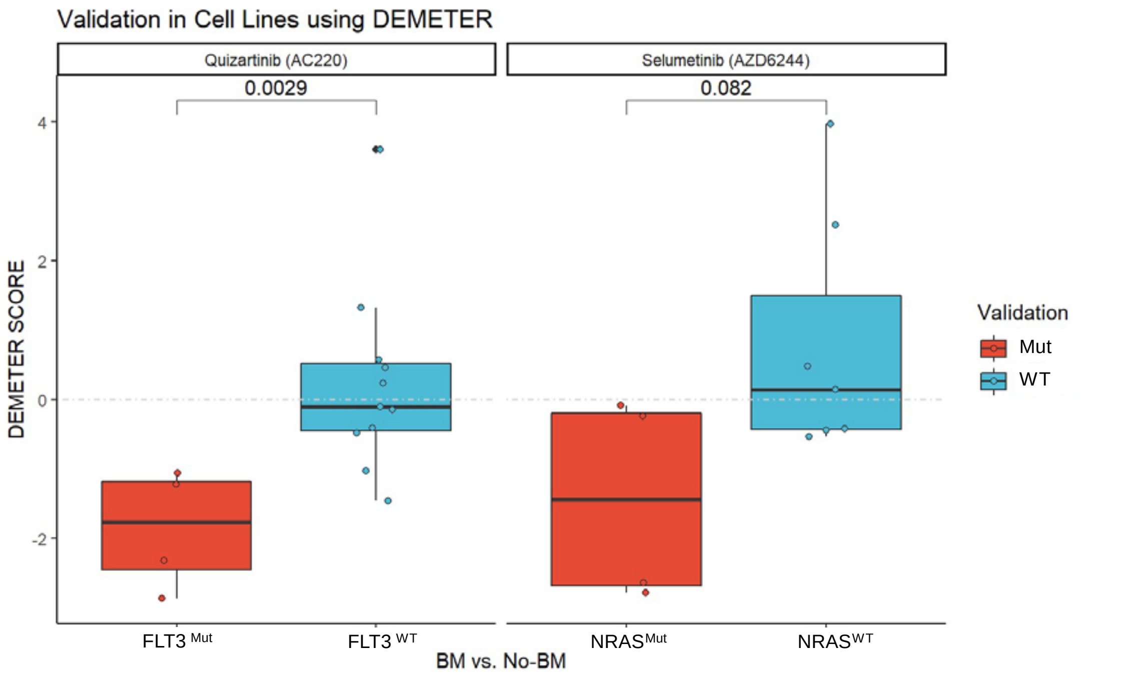

Figure S7: Validation in cell lines using DEMETER Score. In red the cell lines with the biomarker associated with the treatment; in blue the cell lines without the biomarker. On the left, *FLT3*^{Mut} subgroup, on the right *NRAS*^{Mut} subgroup.

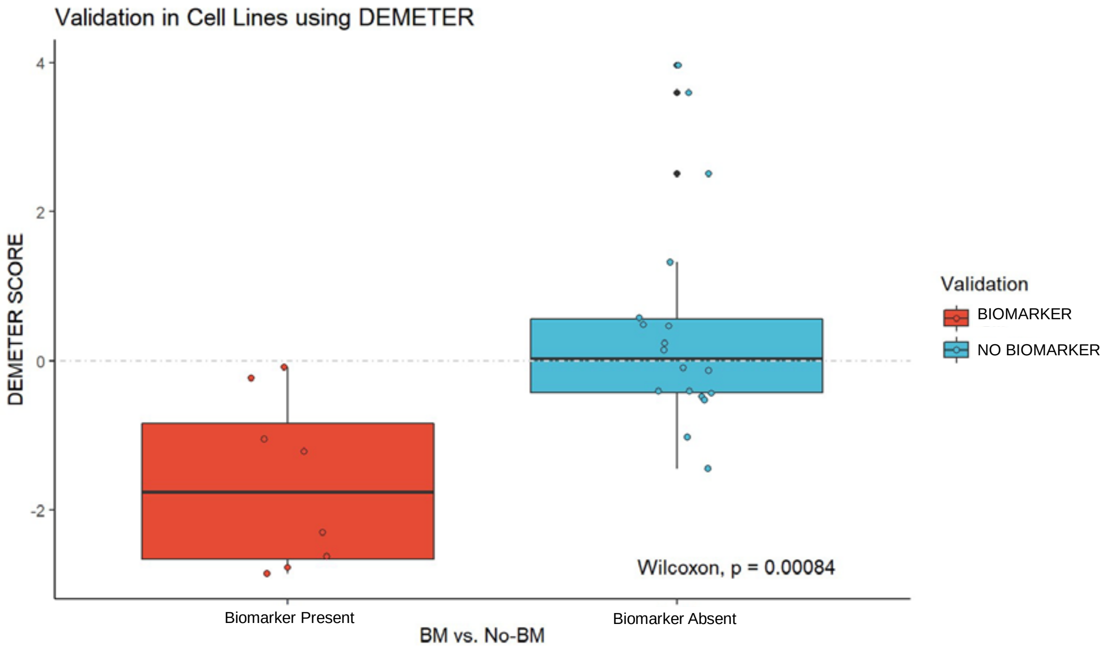

Figure S8: Overall biomarker effect in the DEMETER cohort. All cell lines with the biomarker for the different subgroups are shown in red. In blue are shown all the cell lines that were not having the associated biomarker. P.value was computed using Wilcoxon's Test. The smaller the DEMETER Score is, the more sensitive the cells are.

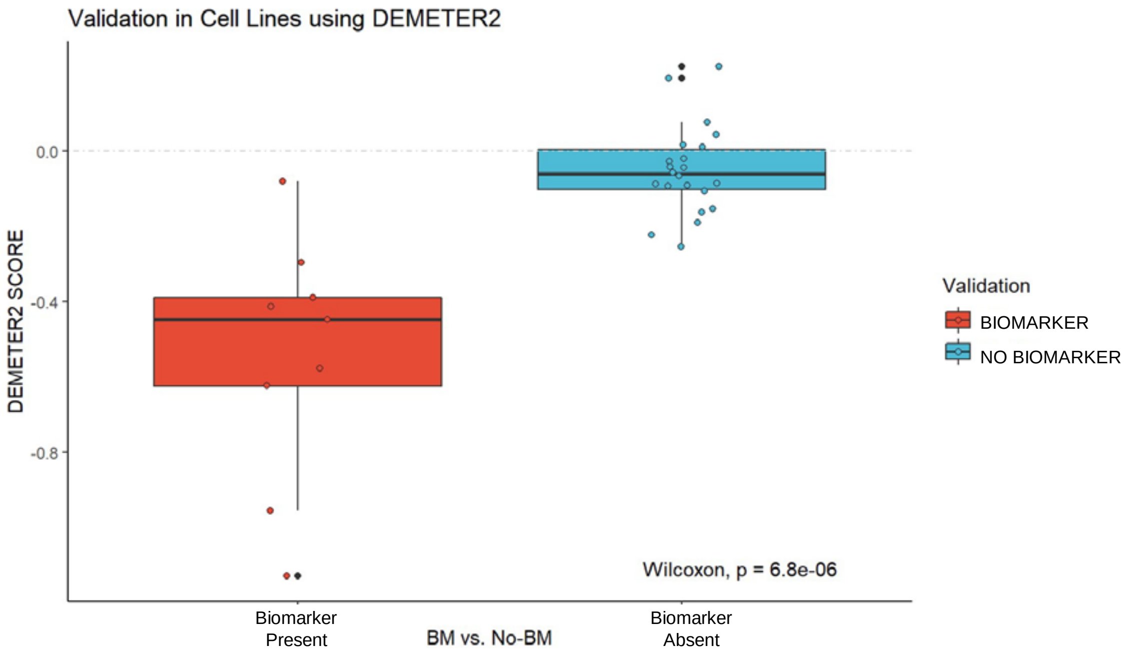

Figure S9: Overall biomarker effect in the DEMETER2 cohort. All cell lines with the biomarker for the different subgroups are shown in red. In blue are shown all the cell lines that were not having the associated biomarker. P.value was computed using Wilcoxon's Test. The smaller the DEMETER2 Score is, the more sensitive the cells are.

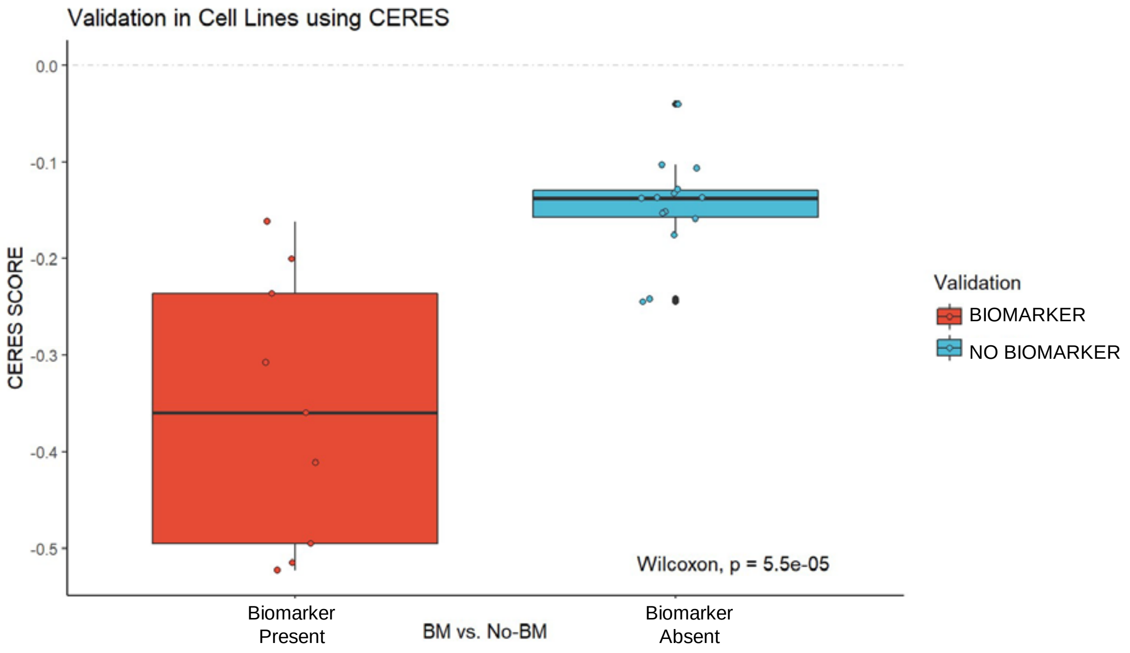

Figure S10: Overall biomarker effect in the CERES cohort. All cell lines with the biomarker for the different subgroups are shown in red. In blue are shown all the cell lines that do not have the associated biomarker. P-value was computed using Wilcoxon's Test. The smaller the CERES Score is, the more sensitive the cells are.

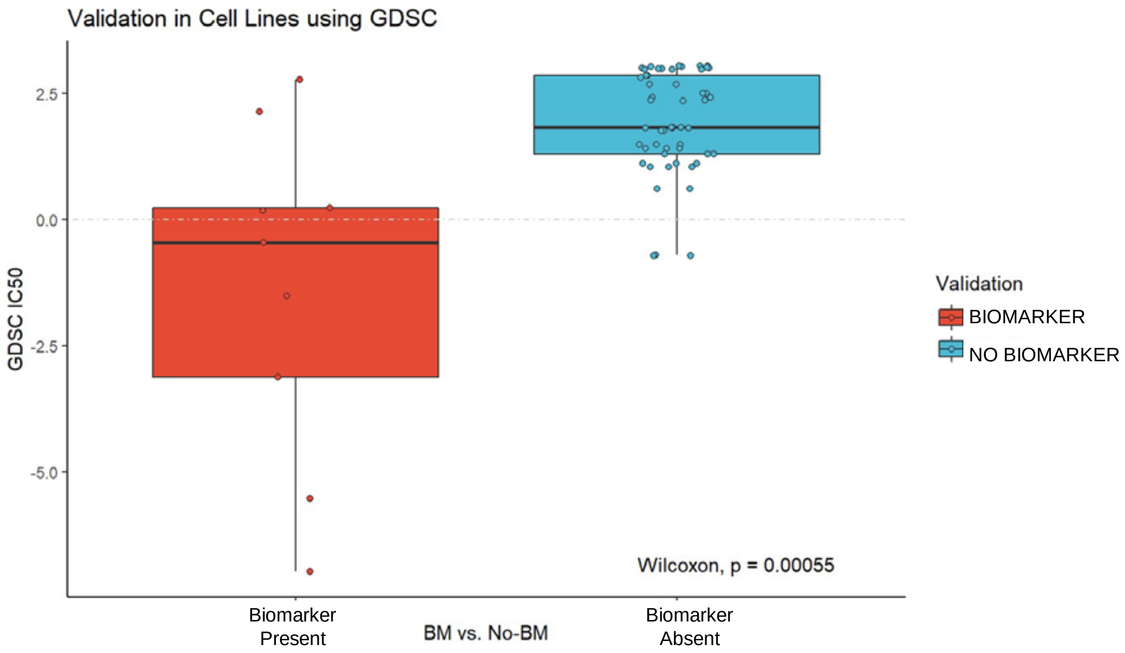

Figure S11: Overall biomarker effect in the GDSC cohort. All cell lines with the biomarker for the different subgroups are shown in red. In blue are shown all the cell lines that were not having the associated biomarker. P.value was computed using Wilcoxon's Test. The smaller the IC_{50} is, the more sensitive the cells are.

Supplementary Results

Standard Classification of Patients

Current patient stratification guides divide AML patients into three subgroups according to their prognosis, namely favorable-, intermediate- and adverse-risk. Each subgroup is defined by a combination of genetic biomarkers that can be either chromosomal rearrangements, genetic mutations, or allele deletions. Thus, the favorable risk subgroup –a 5-year overall survival (OS) of 45% to 80%– includes 45% of AML patients and is diagnosed mainly through the biomarkers *NPM1*^{Mut}, chromosome 16 inversion (inv(16)), and *CEBPA*^{Mut}. The intermediate-risk subgroup –5 years OS of 30%– comprises 25% of AML cases and is associated with the internal tandem duplications in the *FLT3* gene *(FLT3-ITD)*, and *NPM1*^{WT}. Finally, the adverse risk subgroup –5 years OS of 10%– represents 30% of AML cases and has scattered deletions and complex karyotypes as biomarkers[6].

Despite having disparate prognoses, the treatment is similar: all of them are treated with standard induction cytotoxic therapy ("3+7")[6] with different dosages and aggressiveness depending on the severity. Recently, *FLT3* inhibitors have been incorporated as a treatment directed to *FLT3-ITD* patients, but effective treatments for patients who do not have *this* alteration remain an unmet clinical challenge[7]. The lack of treatments directed to *FLT3*^{WT} patients –70% of all AML patients[7]– demands a new classification guide based on the response to therapy based on the presence or absence of specific genetic biomarkers.

BeatAML Treatment Stages

From the 319 patients contained in the study coming from the BeatAML cohort, the chemotherapy phase veries considerabily from the 319, 52 patients had not treatment stage assigned from which 32 were *de Novo* AML. The latter Treatment Stage is shown in the following graph (**Figure S12a**). There are patients who have gone thorugh up to seven different therapy stages. The distribution of the patients accounting for the different chemotherapy phases –excluding the patients with missing info or who did not take any chemotherapy– is the following (**Figure S12b**).

Even though, there could be different agreements mentioning whether it could be more interesting to treat the disease according to the clinical stage: de Novo, Relapsed, Refractory, … Our inclusion criterion focused on the disease itself, as the cohort is an adult cohort with median of age 61, the results of the manuscript reflect an approach to treat the adult AML disease, independently from the chemotherapy phase.

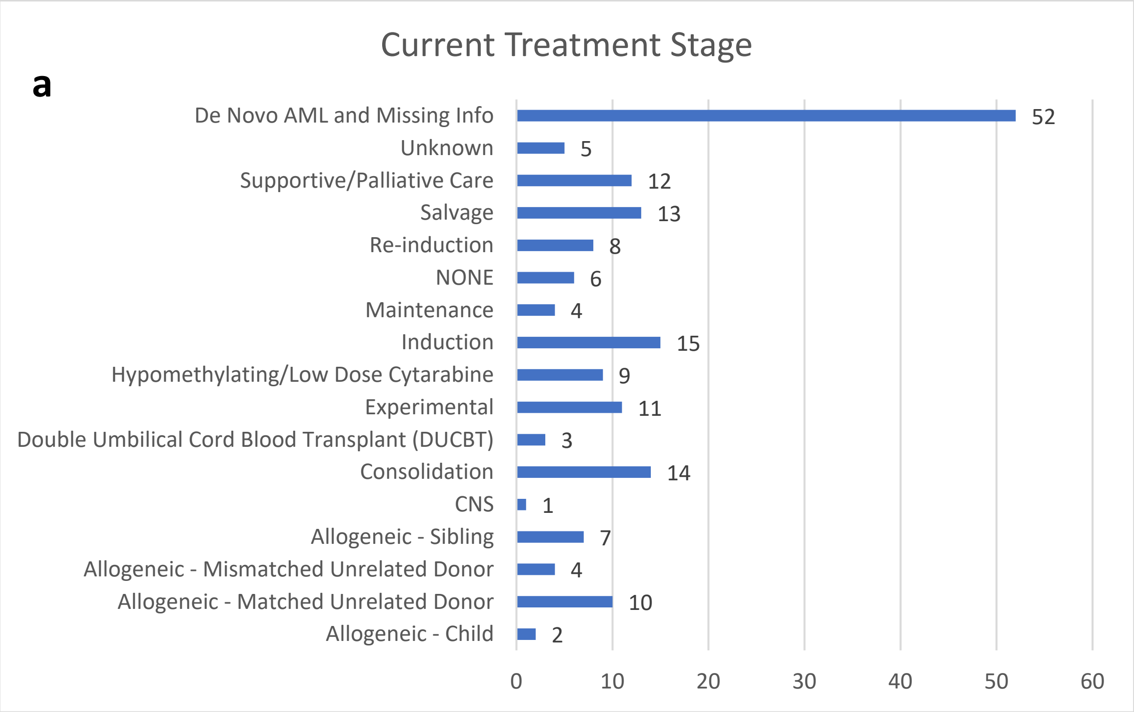

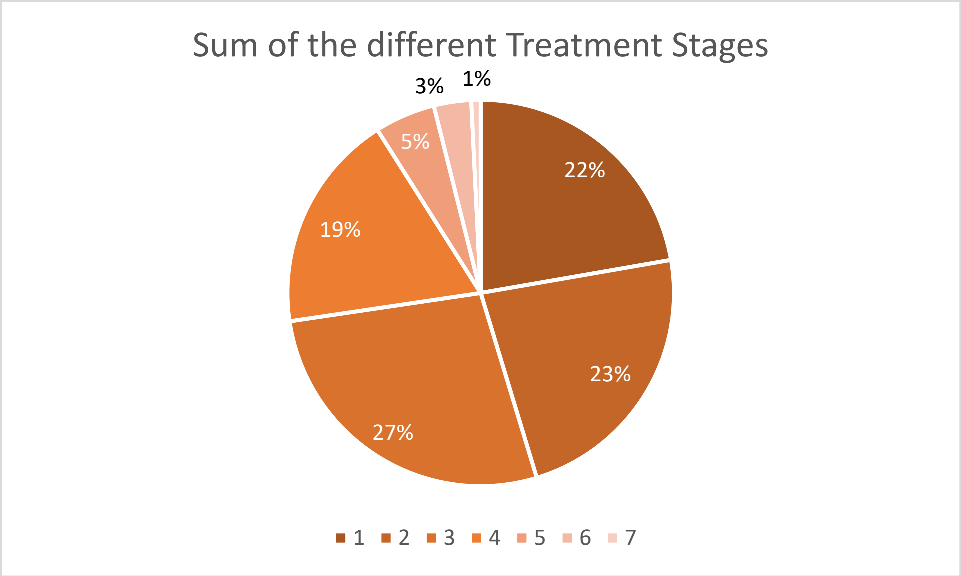

Figure S12: Treatment Stages in the BeatAML cohort. A) From the 319 patients contained in the study coming from the BeatAML cohort, the chemotherapy phase veries considerabily. B) Distribution of the patients accounting for the different chemotherapy phases excluding the patients with missing info or who did not take any chemotherapy.

Biomarker Analysis

We performed an extensive biomarker analysis to characterize the WES from Beat AML[8] cohort using the maftools[9] R package (version 2.10.05). We intended to understand the different processes that are regulating the biomolecular characterization of this cohort. To do it, we plotted several figures that contain the relevant information concerning the genetic profile of the patients in the cohort.

Figure S13 shows that 88.16% of patients have at least one genetic variation, the majority of these variants are missense, and *DNMT3A*, *NPM1* and *NRAS* are the most commonly mutated genes in this cohort. Moreover, from these variants, the majority correspond to single Nucleotide Variants (SNVs) with the signature C>T that is quite frequent in malignant cancer types followed by C>A which is associated with environmental exposure[10] (**Figure S14**). We appreciated that the median of genetic variants per patient is 8 variants and that only *FLT3* and *SRSF2* had insertions i.e. *FLT3-ITD*.

We tried to understand more in-depth the SNVs changes and classified them into transitions (two-ring purines or one-ring purines changes) and transversions (changes of purines for pyrimidines) we discovered that in this cohort the transitions are more frequent with the most common transition being C>T, followed by a transversion C>A (**Figure S15**).

Using the Whole Exome Sequencing (WES) data provided in the Beat AML cohort we analysed co-occurrence and mutual exclusivity of genetic variants at the gene level. We appreciated that *FLT3* and *NPM1* variants are co-occurrent (p-value <0.05), and *FLT3* and *TP53* (p-value <0.05) and NRAS and IDH2 are mutually exclusive respectively (p-value <0.05) (**Figure S16**).

AML is a highly heterogenetic disease and consequently, genetic translocations are included as possible biomarkers. All translocations can be identified by a gene fusion product: inv(16) with *CBFβ-MYH11*, inv(3) with *RPN1-EVI1*, t(9;11) with *MLLT3-MLL*, and t(8;21) with *RUNX1-RUNX1T1*. From these translocations, inv(16) appears in co-occurrence with *FLT3*, *KIF20B*, and *ADAMTS7* variants. Whereas t(9;11) can appear with *NRAS* variants and inv(3) with *KIT* variants (**Figure S17**).

Finally, we addressed the biological consequences of this mutational landscape by interrogating the alteration of the most common oncogenic pathways (**Figure S18**). We saw that RTK-RAS is the most affected pathway, having an alteration in 31 out of 85 genes and it is present in 237 out of 608 patients. Remarkable alterations include NOTCH, WNT, MYC, TP53 and TGF-β pathways. We included a summary by the patient showing the complete pathway alterations (**Figure S19**).

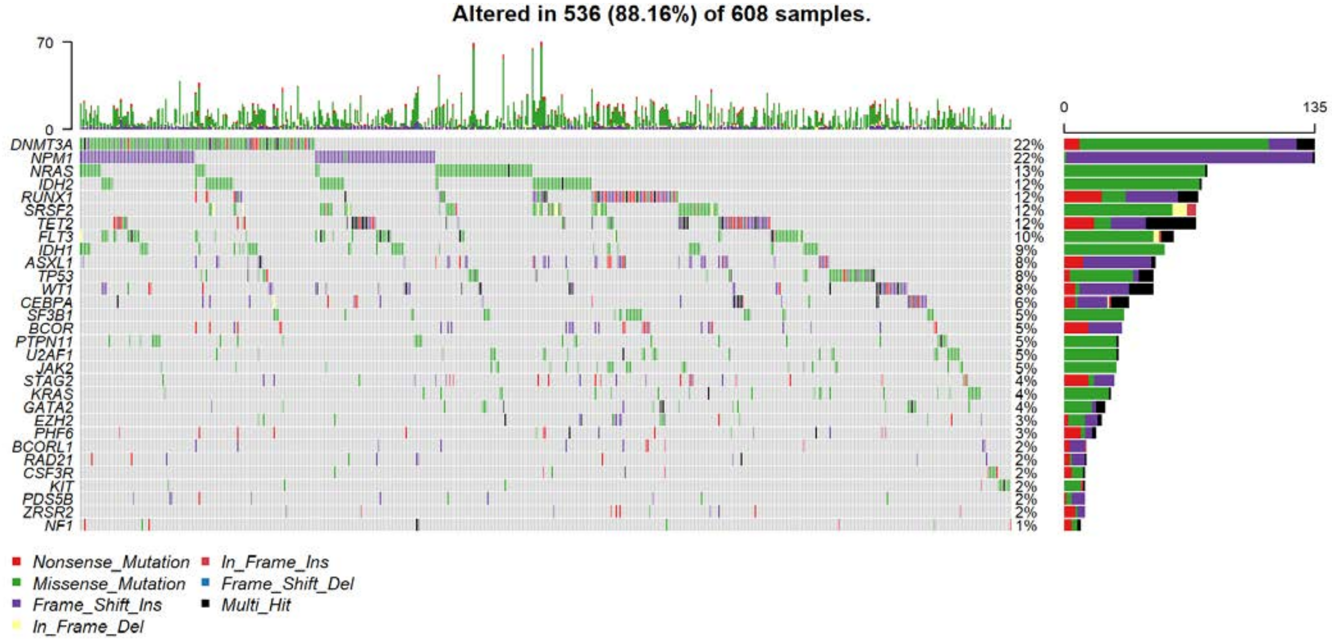

Figure S13: Mutational Status of Beat AML cohort. This plot shows the different types of genetic variants, the lateral barplot shows the sum of all the genetic alterations in all patients and colours the type of variant. In the horizontal axis we have the individual patients' information, showing some patients having up to 70 co-occurring mutations. Of the 608 patients, 538 had at least one genetic variant (88.16%).

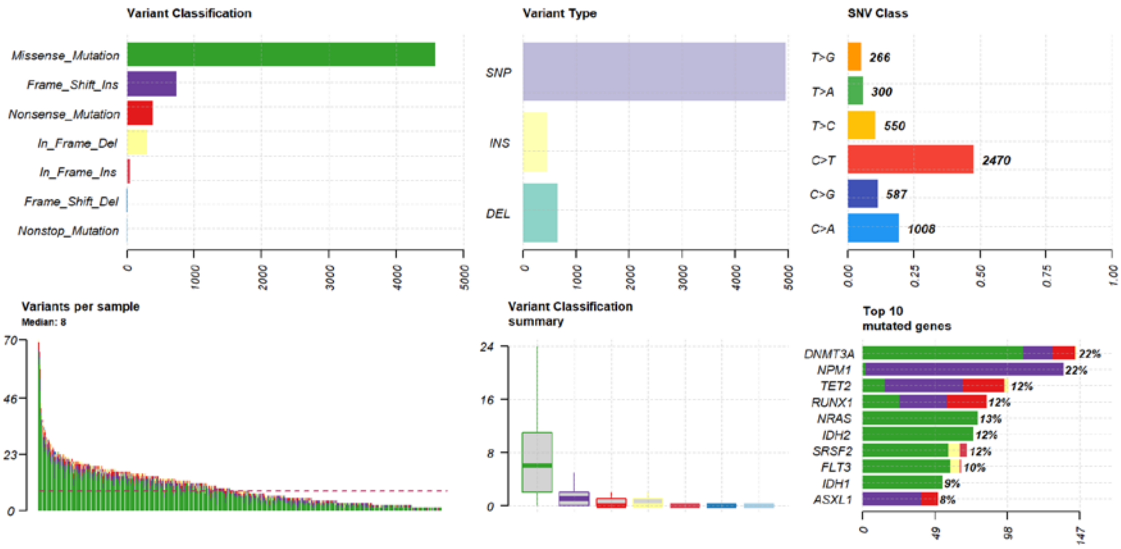

Figure S14: Genetic Variant Type Summary. Variant classification plot represents the different genetic variant types, in terms of functionality and we see that the most common is missense variant, the Variant type plot represents the type of structural variant, whether they are Single Nucleotide Variants (SNVs) or Indels with a clear predominance of SNVs, SNV classification plot shoed the type of mutational signature that is predominant with the signature C>T that is quite frequent in malignant cancer types followed by C>A which is associated with environmental exposure[10]. The Variants per Sample Plot, tells that there is a median of 8 variants per patient. The variant classification summary plot summarises all the prior plots and, finally, the Top 10 mutated genes plot, shows for each of the top 10 genes the type of mutation.

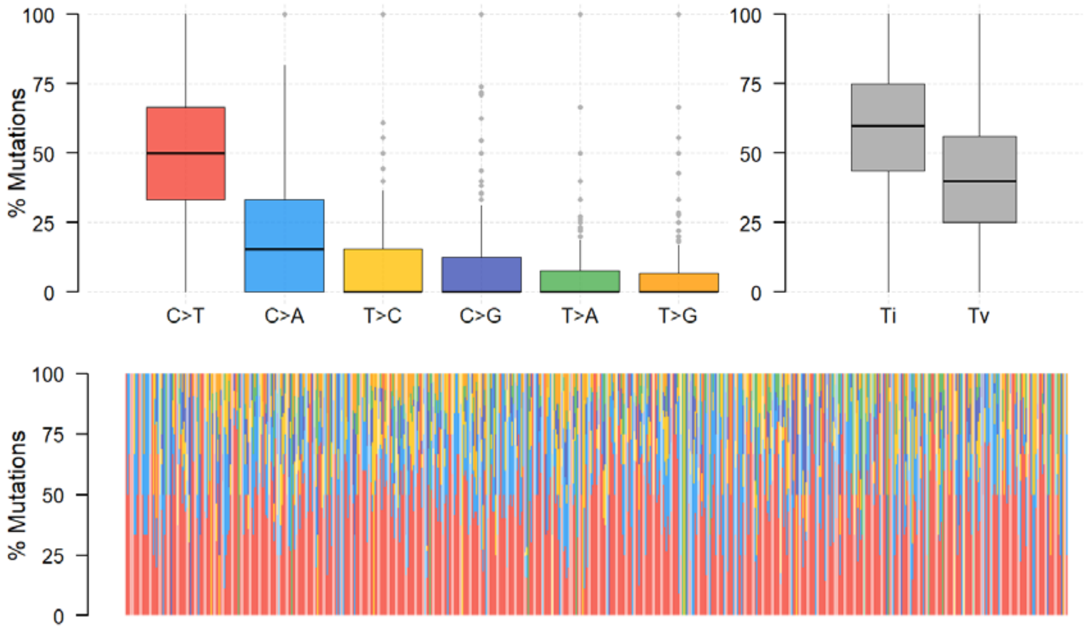

Figure S15: Transitions(Ti) and Transversions (Tv) landscape in Beat AML cohort. Ti vs. Tv plot shows the number of the transitions and transversions showing that even when transversions seem most probable to occur, transitions are more present in this cohort. Boxplot showing overall distribution of six different conversions and as a stacked barplot showing the fraction of conversions in each sample. The most common transition is C>T, followed by a transversion of C>A.

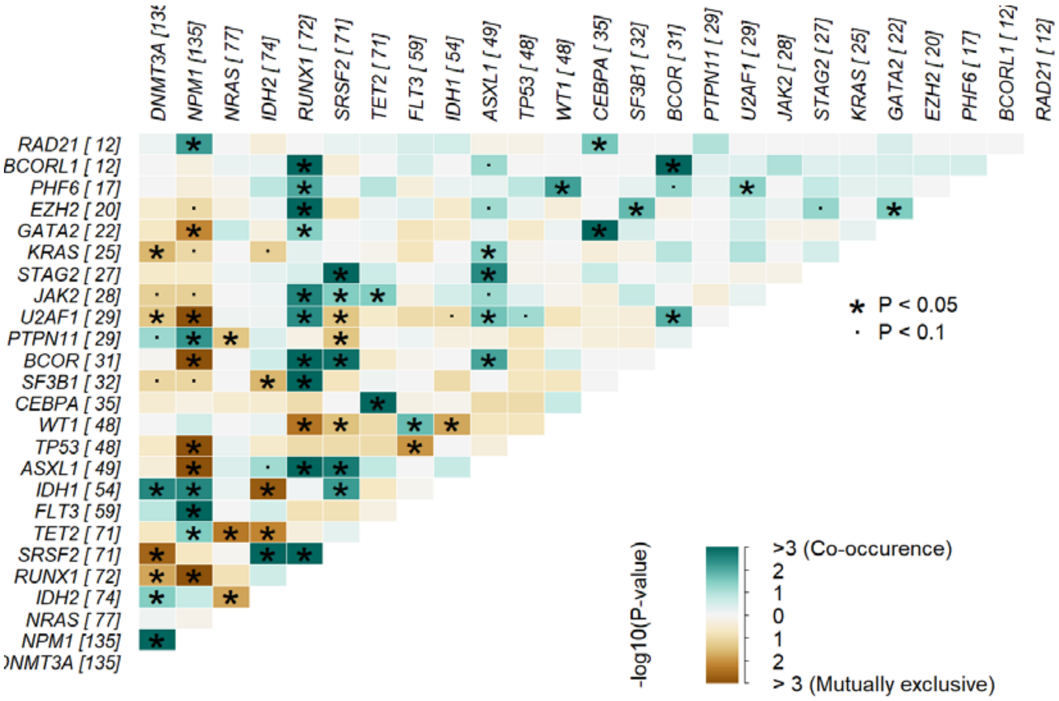

Figure S16: Somatic Interactions. Mutually exclusive or co-occurring set of genes calculated using pair-wise Fisher's Exact test. Associations plotted in green represent Co-occurrence while brown is a sign of mutual exclusivity. Stars are assigned to associations with P< 0.05. We appreciated that FLT3 and NPM1 variants are co-occurrent, and FLT3 and TP53 and NRAS and IDH2 are mutually exclusive respectively.

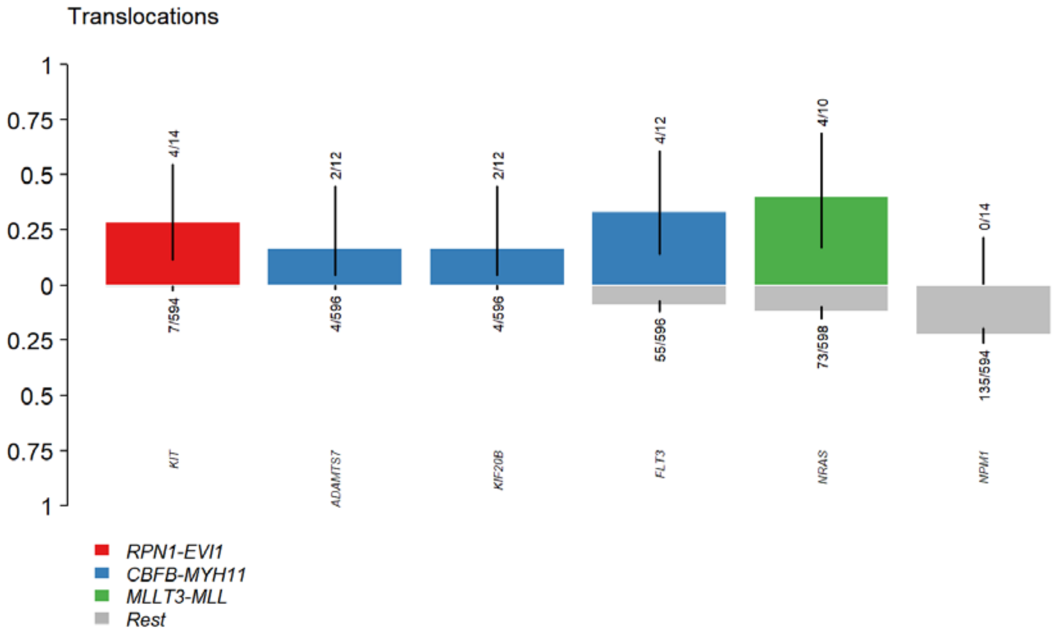

Figure S17: Translocations and SNVs. All translocations can be identified by a gene fusion product: inv(16) with *CBFβ-MYH11*, inv(3) with *RPN1-EVI1*, t(9;11) with *MLLT3-MLL*, and t(8;21) with *RUNX1-RUNX1T1*. From these translocations, inv(16) appears in co-occurrence with *FLT3*, *KIF20B*, and *ADAMTS7* variants. Whereas t(9;11) can appear with *NRAS* variants and inv(3) with *KIT* variants

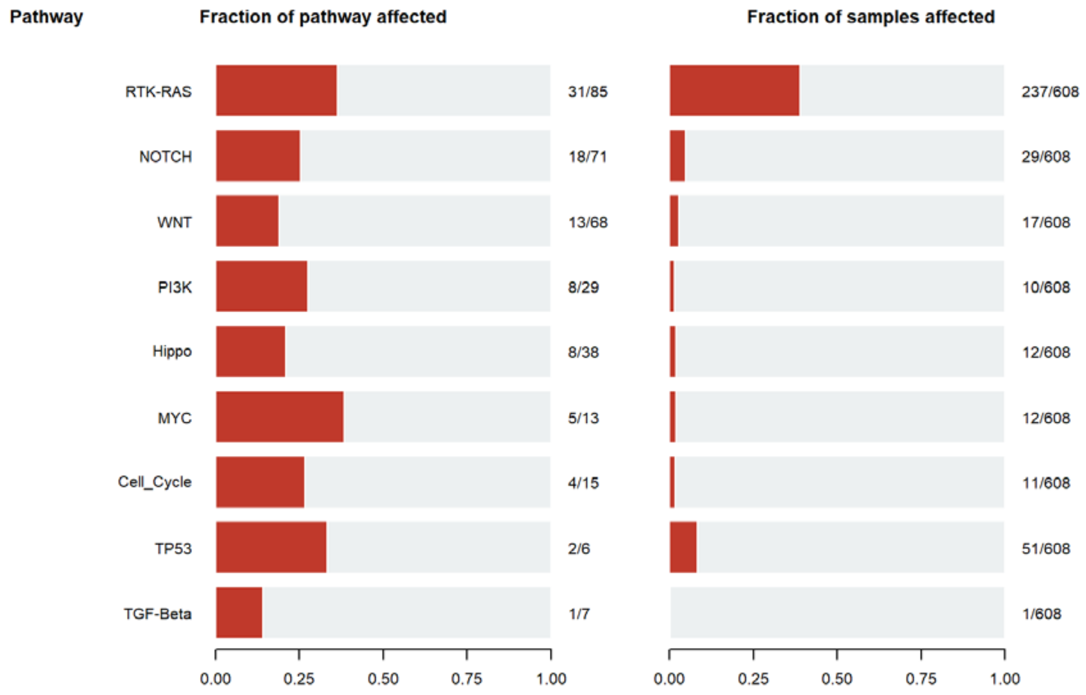

Figure S18: Oncogenic Signalling Pathways altered in Beat AML cohort. The barplot on the left represents the proportion of genes that are altered in the pathway, whereas the barplot on the right, shows the proportion of samples that are having an alteration in that pathways.

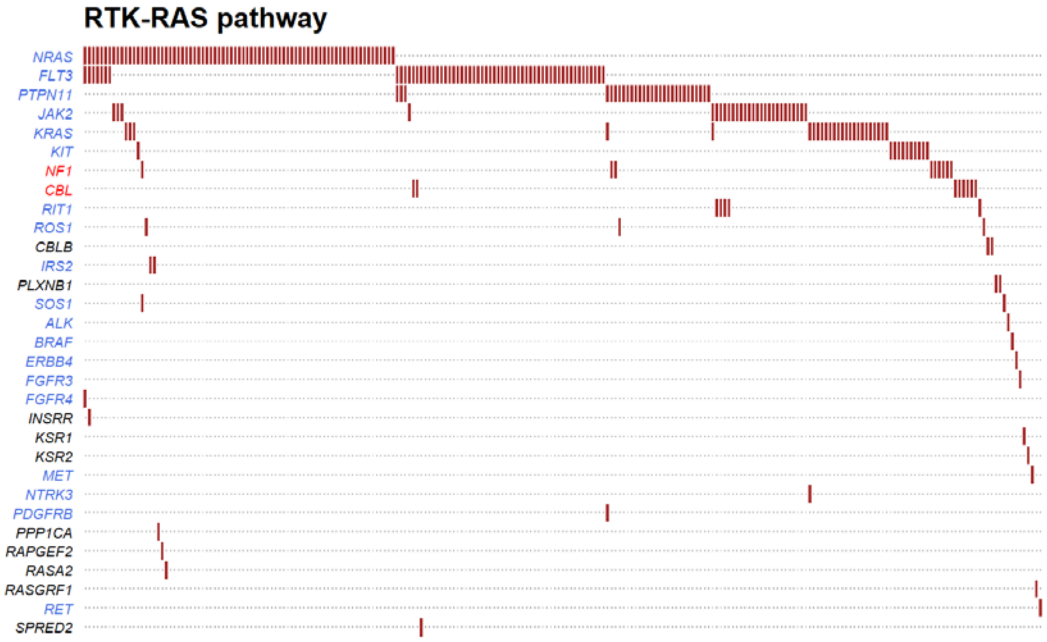

Figure S19: RTK-RAS pathway alterations. In the Y-axis all genes are included in the pathway, in blue the oncogenes and red tumour suppressor genes. In the X-axis all the samples with RTK-RAS altered and the red marks show the pathway genes altered for each sample.

Individual Drug Biomarker Associations

Drugs provided by the ex-vivo experiments in the Beat AML cohort respond to the following diseases categories (**Figure S20**).

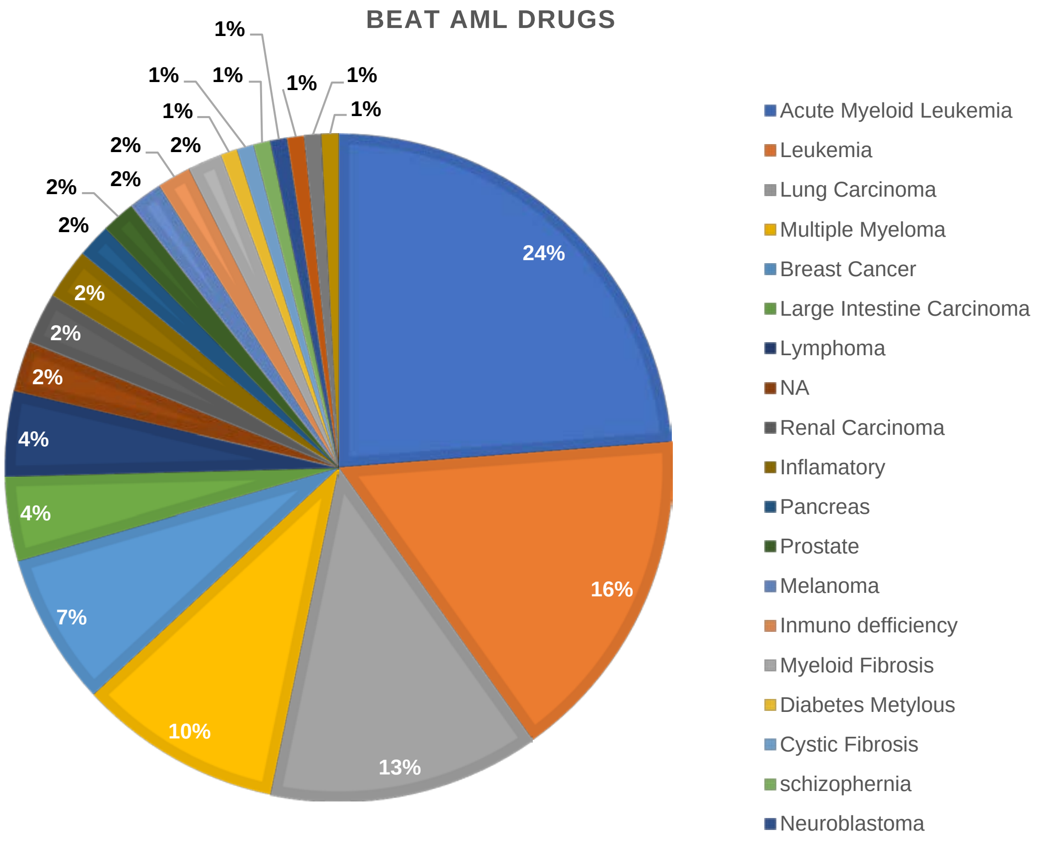

Figure S20: Beat AML Drugs distribution. Drugs studied in the BeatAML cohort cover a wide variety of different cancers and diseases, from which 24% are designed, experimental, or already prescribed for AML, 16% for other leukemias, 10% are shown concerning multiple myeloma, and 4% for different types of lymphomas. This means that 54% of the drugs have been studied for hematological malignancies.

Once we performed the pre-processing in the gene variants data and drug screenings, we obtained the individual associations as mentioned in the main manuscript. Individual associations show how biomarkers can influence sensitivity or resistance for a certain drug. We discovered a total number of 156 significant associations (**Table S1**), all of them, candidates for patient stratification. **Figure S21** contains some relevant examples.

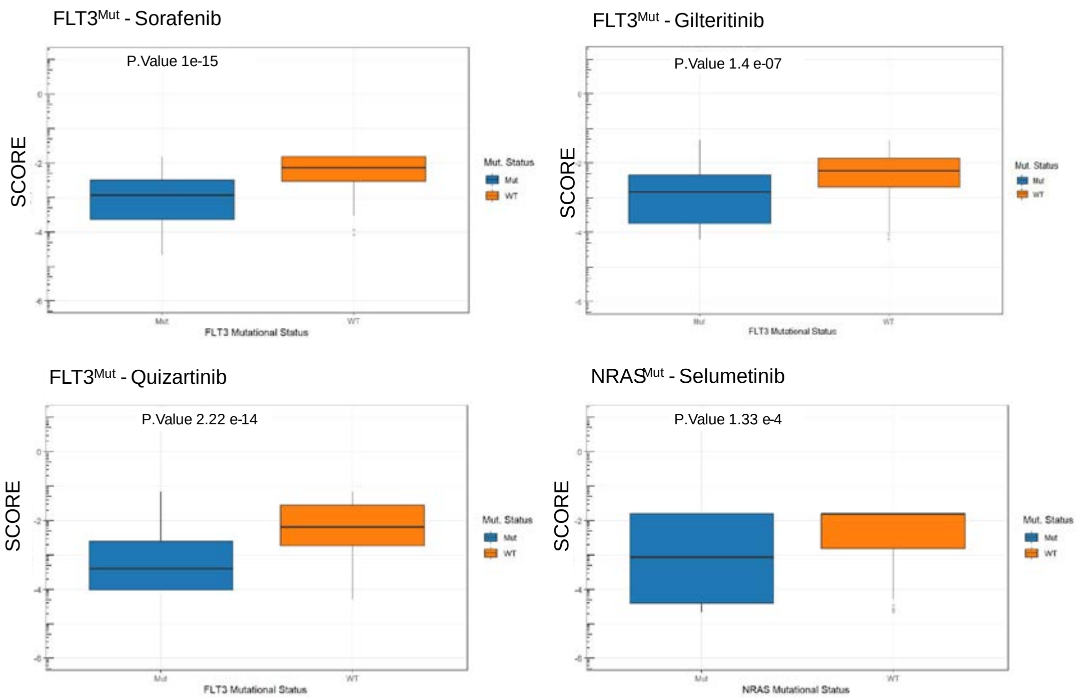

Figure S21. Relevant Individual Drug-Biomarker Associations. In blue the patients with the biomarker in orange the patients without the biomarker. P-value association is corrected according to the main manuscript. The score shows the differential IC_{50}, more negative means more sensitive.

Individual Analysis of the Predicted Subgroups

We tried to understand more in-depth each of the subgroups whose treatment according to MOM is different. The methodology is described in the Methods section of the main manuscript. This section includes the results from the enrichment analysis based on gene expression including all the functions that appeared as statistically enriched from the two conditions up and downregulated. The color in the plots refers to the statistical significance the redder it is the more significant, the bluer it is, the less significant it is. **Figures S22-S29**, describe all these conditions showing a bar plot, dot plot, upset plot, and emap plot to better understand the relevance and interaction of the obtained figures.

Figure S22: FLT3^{Mut} – Quizartinib Upregulated GO enrichment Functions

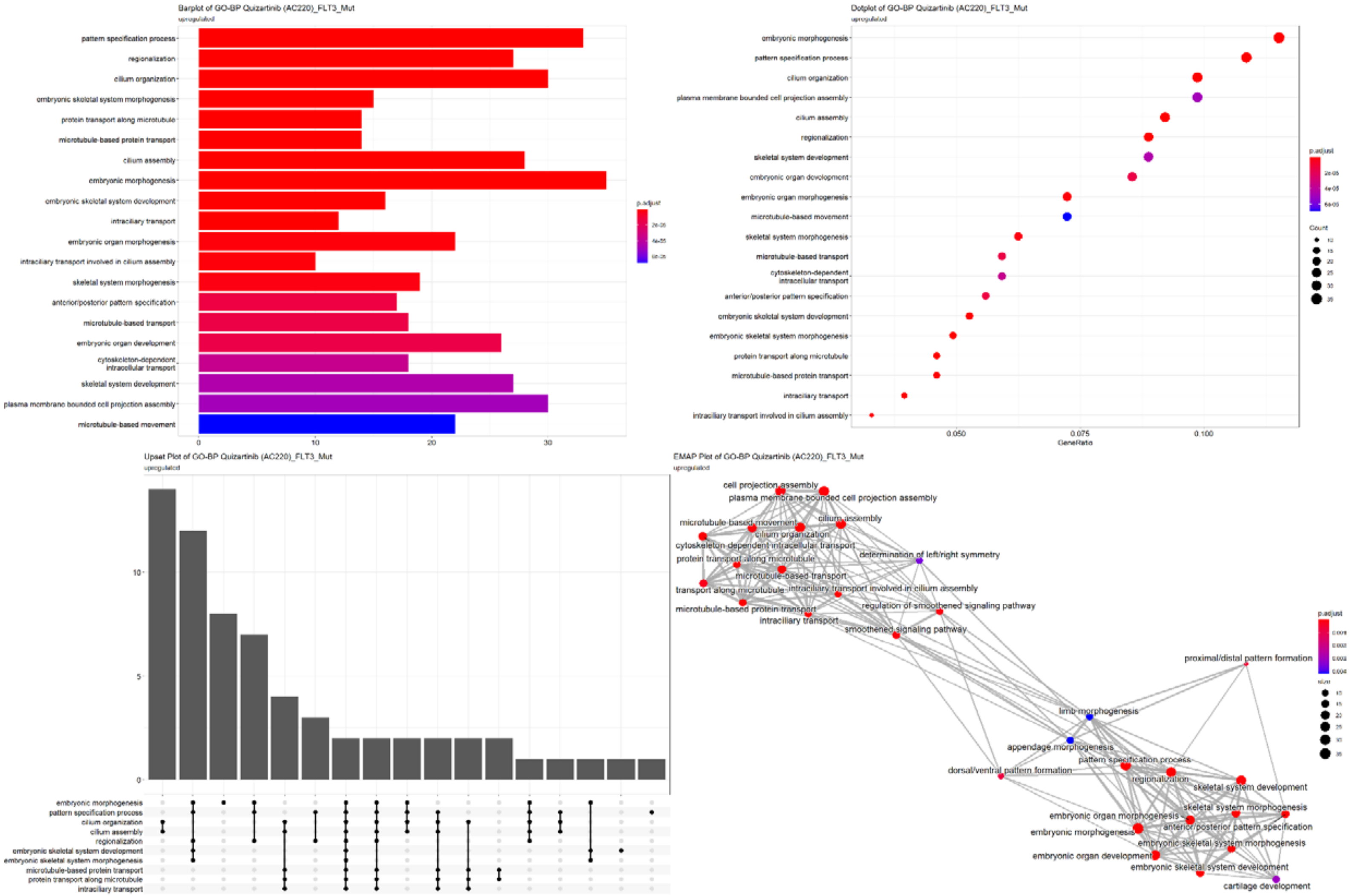

Figure S23: FLT3^{Mut} - Quizartinib downregulated GO enrichment Functions

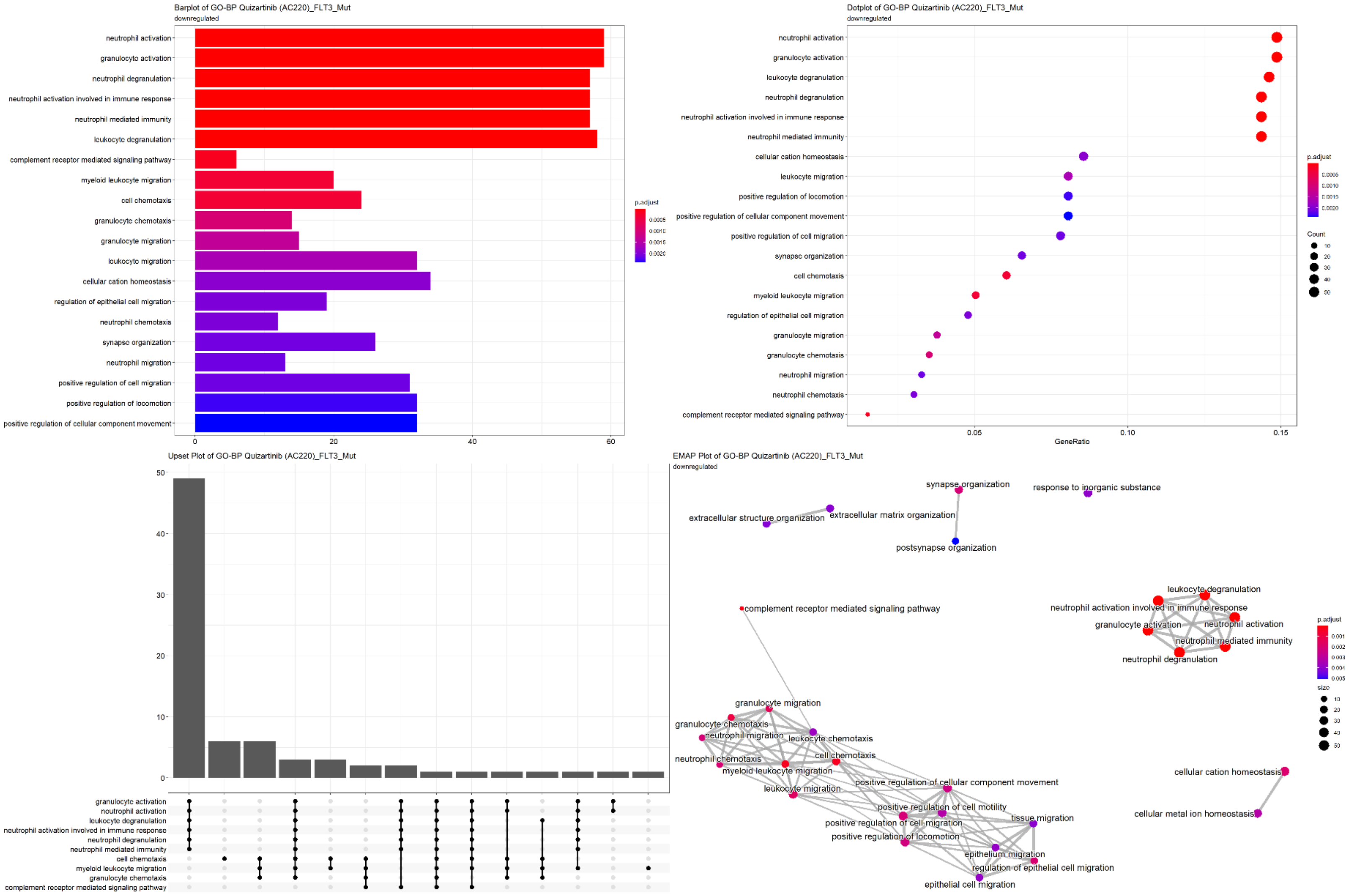

Figure S24: FLT3^{WT} & Inv(16) – Trametinib GO upregulated enrichment Functions

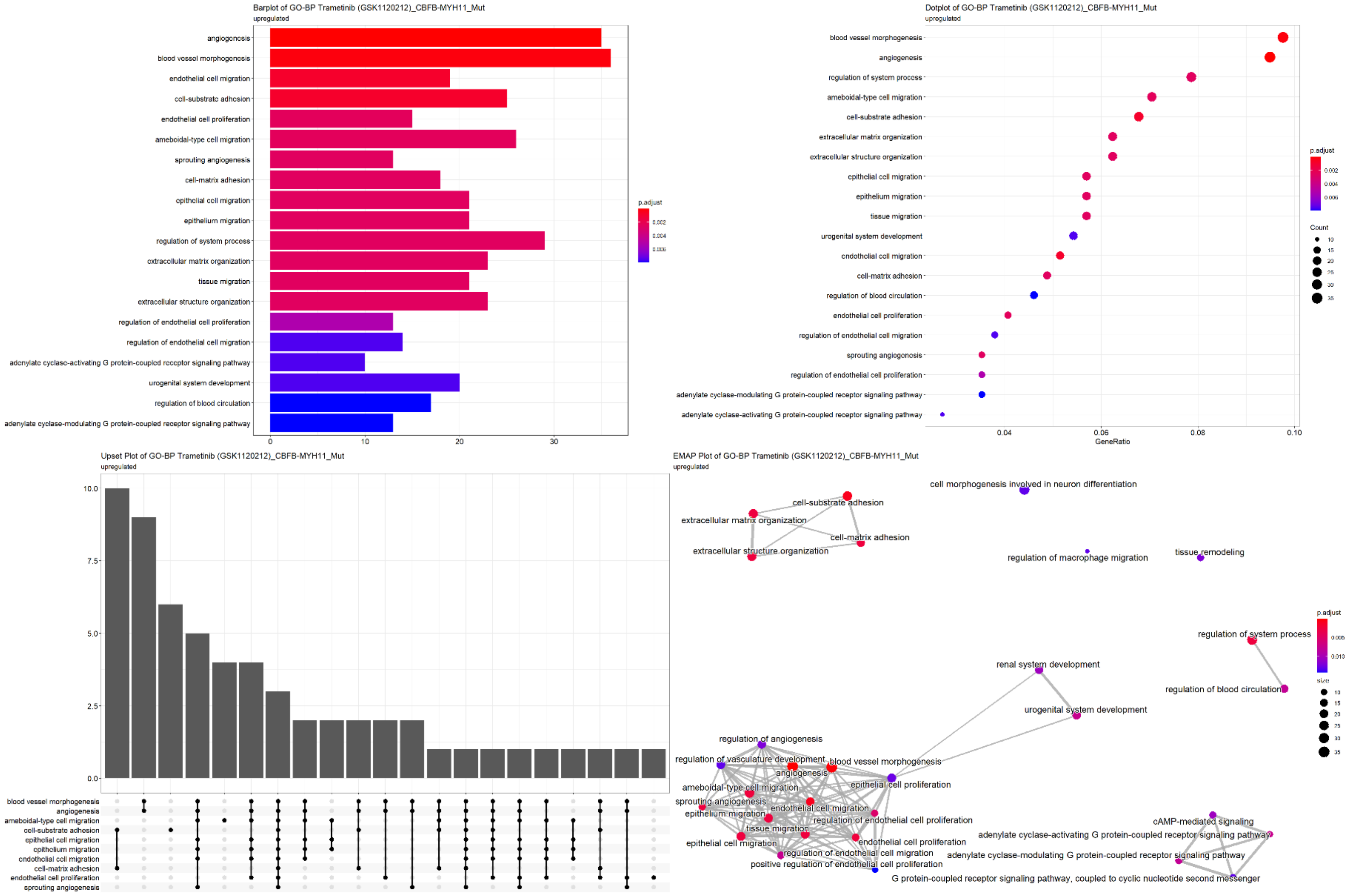

Figure S25: FLT3^{WT} & Inv(16) – Trametinib GO downregulated enrichment Functions

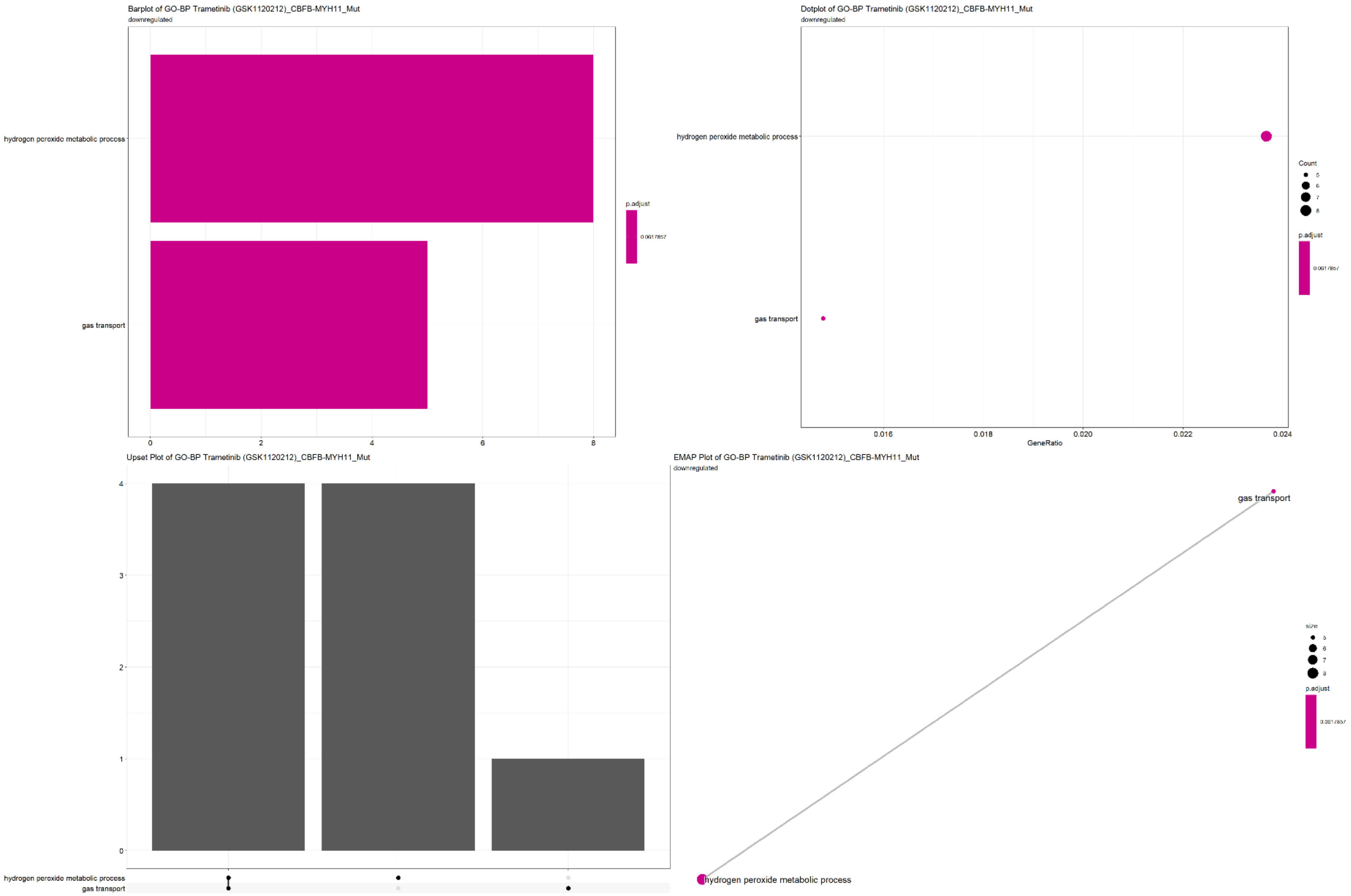

Figure S26: FLT3^{WT} & no Inv(16) & NRAS^{Mut} – Selumetinib GO upregulated enrichment Functions

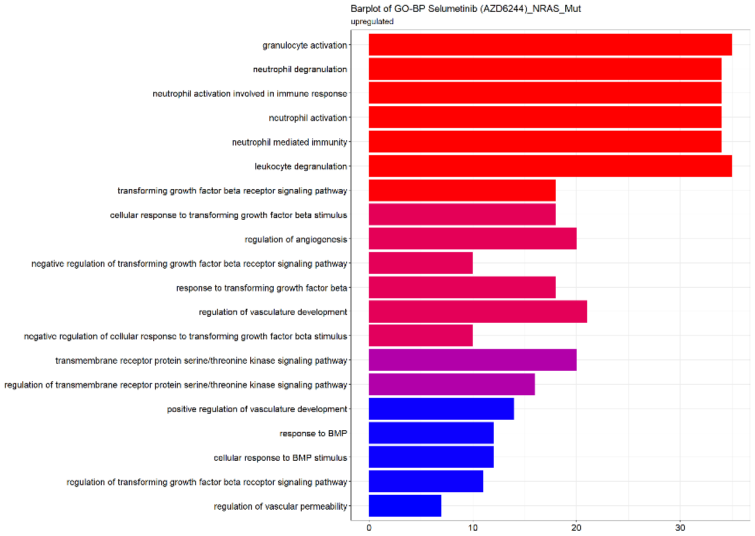

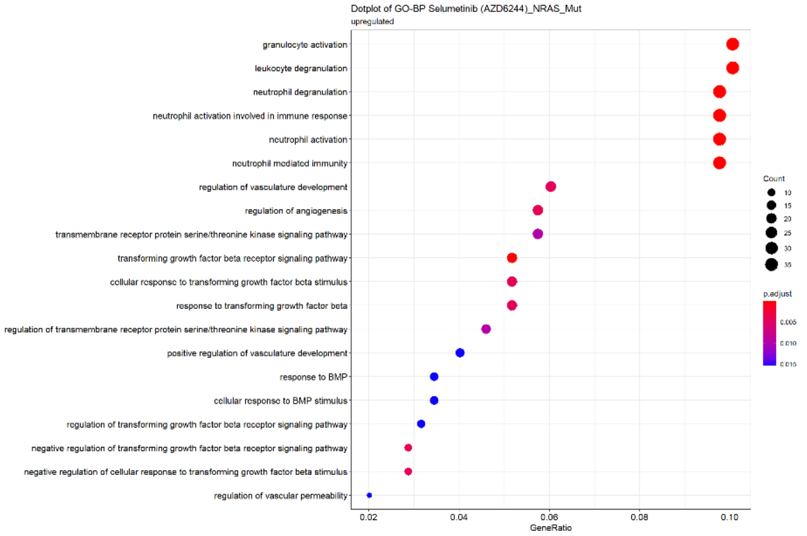

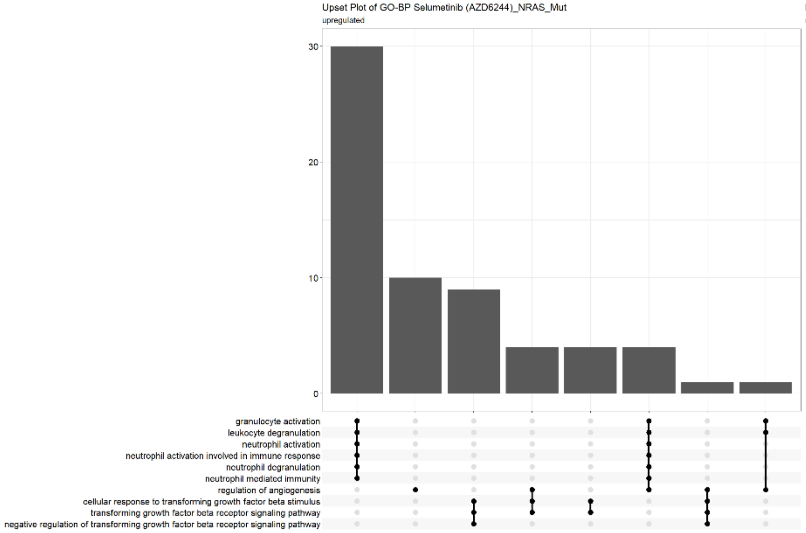

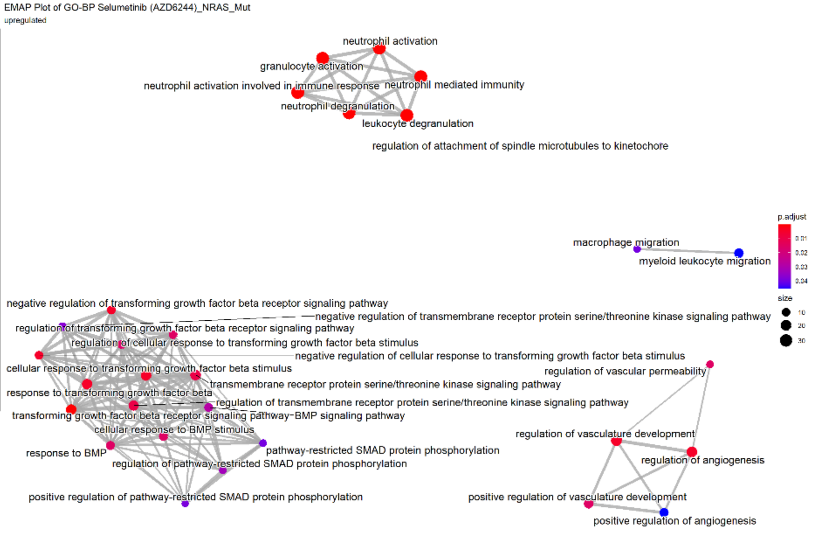

Figure S27: FLT3^{WT} & no Inv(16) & NRAS^{Mut} – Selumetinib GO downregulated enrichment Functions

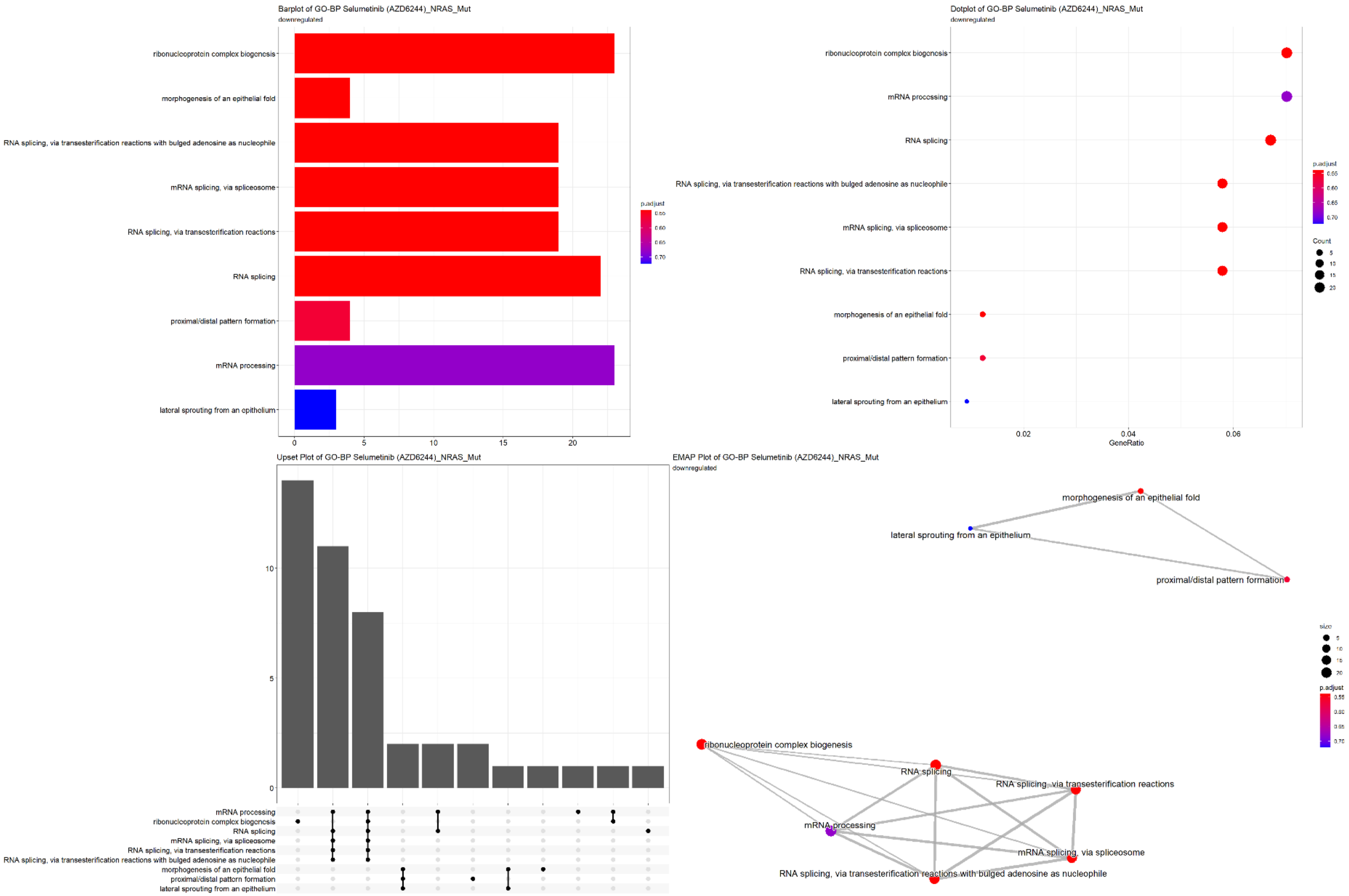

Figure S28: Rest – Crizotinib GO upregulated enrichment Functions

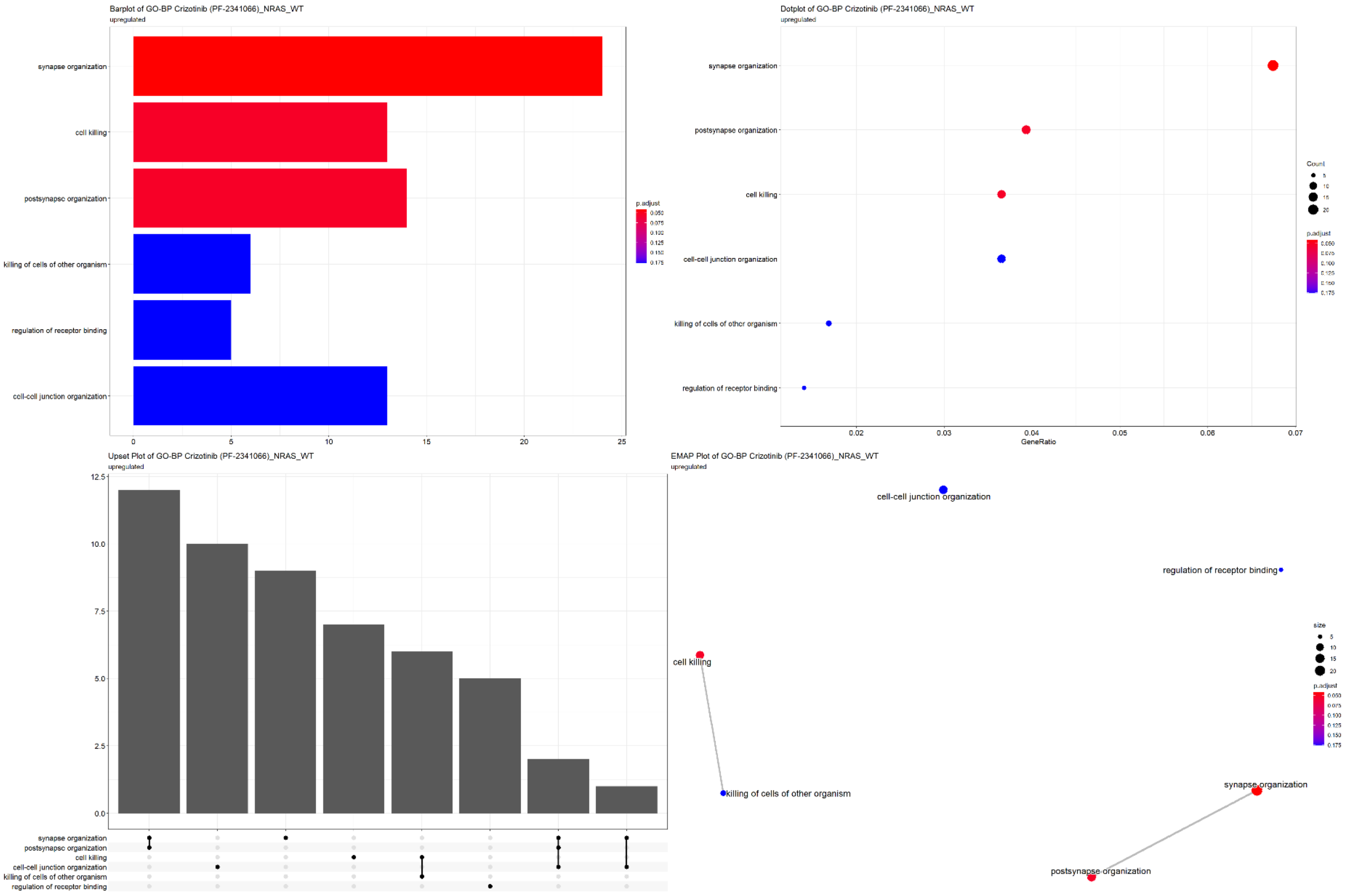

Figure S29: Rest – Crizotinib GO downregulated enrichment Functions

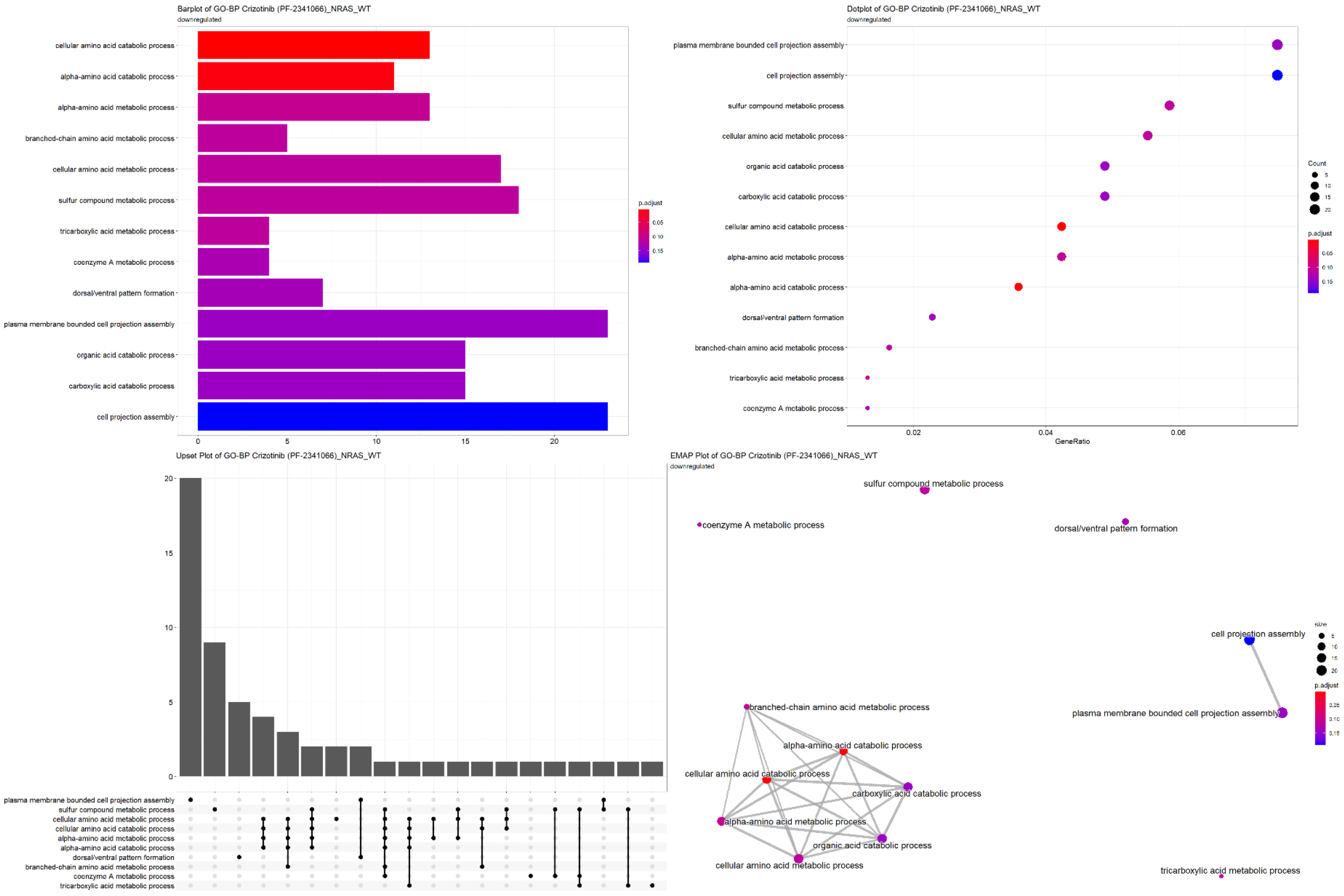

References

1. Hastie, T., Tibshirani, R., Narasimhan, B. & Gilbert, C. impute: Imputation for microarray data. *Bioinformatics* **17**, 520–525 (2001).

2. JJ Allaire, Kevin Ushey, Yuan Tang, and D. E. reticulate: R Interface to Python. (2017).

3. McFarland, J. M. *et al.* Improved estimation of cancer dependencies from large-scale RNAi screens using model-based normalization and data integration. *Nat. Commun.* **9**, 1–13 (2018).

4. Meyers, R. M. *et al.* Computational correction of copy number effect improves specificity of CRISPR-Cas9 essentiality screens in cancer cells. *Nat. Genet.* **49**, 1779–1784 (2017).

5. Yang, W. *et al.* Genomics of Drug Sensitivity in Cancer (GDSC): a resource for therapeutic biomarker discovery in cancer cells. *Nucleic Acids Res.* **41**, D955–D961 (2013).

6. Zeisig, B. B., Kulasekararaj, A. G., Mufti, G. J. & Eric So, C. W. SnapShot: Acute myeloid leukemia. *Cancer Cell* **22**, (2012).

7. Sutamtewagul, G. & Vigil, C. E. Clinical use of FLT3 inhibitors in acute myeloid leukemia. *Onco. Targets. Ther.* **Volume 11**, 7041–7052 (2018).

8. Tyner, J. W. *et al.* Functional genomic landscape of acute myeloid leukaemia. *Nature* **562**, 526–531 (2018).

9. Mayakonda, A., Lin, D. C., Assenov, Y., Plass, C. & Koeffler, H. P. Maftools: Efficient and comprehensive analysis of somatic variants in cancer. *Genome Res.* **28**, 1747–1756 (2018).

10. Alexandrov, L. B. *et al.* The repertoire of mutational signatures in human cancer. *Nature* **578**, 94–101 (2020).
